# Dynamics of Marine Debris Ingestion by Profitable Fishes Along The Estuarine Ecocline

**DOI:** 10.1038/s41598-019-49992-3

**Published:** 2019-09-18

**Authors:** Guilherme V. B. Ferreira, Mario Barletta, André R. A. Lima, Simon A. Morley, Monica F. Costa

**Affiliations:** 1 Laboratory of Ecology and Management of Coastal and Estuarine Ecosystems, Department of Oceanography, Federal University of Pernambuco (UFPE). Av. Arquitetura S/N, Cidade Universitária, Recife, Pernambuco CEP: 50740-550 Brazil; 2 British Antarctic Survey, Natural Environment Research Council, High Cross, Madingley Road, Cambridge, CB30ET UK

**Keywords:** Wetlands ecology, Ecosystem ecology

## Abstract

The dynamics of microfilament (<5 mm) ingestion were evaluated in three species of snooks. The ingestion of different colours and sizes of microfilaments were strongly associated with the spatio-temporal estuarine use and ontogenetic shifts of snooks. Their feeding ecology was also analysed to assess dietary relationships with patterns of contamination. All species were highly contaminated with microfilaments. The highest ingestion of microfilaments occurred in the adults, when fishes became the main prey item and also during the peak of fishing activities, in the rainy season. This suggests that trophic transfer, in addition to periods of high availability of microfilaments are important pathways for contamination. The ingestion of microfilaments of different colours and sizes was likely influenced by input sources. Blue microfilaments were frequently ingested, and appear to have both riverine and estuarine inputs, since they were ingested in all seasons and habitats. Purple and red microfilaments were more frequently ingested in the lower estuarine habitats. The length of microfilaments was also associated with environmental variability. Longer microfilaments were ingested in habitats with greater riverine influence, the opposite was observed for shorter microfilaments. Therefore, microfilament contamination in snooks are a consequence of their ecological patterns of estuarine uses through different seasons and life history stages.

## Introduction

Marine debris are among the greatest environmental concerns of the XXI century, especially because they are ubiquitous contaminants of a range of aquatic ecosystems^1–3^. Moreover, the global production of plastics, one of the most common marine debris, is increasing, with annual productions exceeding 300 million tonnes^4^. Plastics are widely used in industry and domestically, with little prospect of their use decreasing as they are a versatile, cheap and durable material^5^.

The introduction of debris into the aquatic environment occurs by accident or intentionally by improper disposal practices, such as illegal dumping of sewage and solid wastes into rivers and oceans^6^. Additionally, the fishing industry is recognized as one of the major sources of marine debris, responsible for the introduction of tonnes of items as the result of *in situ* maintenance, abrasion and environmental exposure of fishing gears^7,8^.

Once in the aquatic environment, marine debris tend to breakdown, into smaller particles (<5 mm) due to the weathering processes caused by hydrodynamic forces and photodegradation^8,9^. Because of their diminutive size, small particles of marine debris are more likely to be ingested by marine biota. Indeed, marine debris ingestion has been reported in a wide variety of taxa, from planktonic to nektonic species^10,11^, and studies have reported high contamination rates, with more than 60% contamination of fishes caught in field surveys^12,13^. The high concentrations and wide distribution of marine debris means that they can interact with every trophic guild, being directly ingested and transferred across trophic levels, which can explain the resulting high contamination rates found in top predator fishes^14,15^. One of the strongest links between marine wildlife and humans are through top predators, and so marine debris may indirectly affect human populations when these resources are consumed^16,17^.

Marine debris can affect wildlife through chemical transfer of adsorbed organic and inorganic pollutants^18^. It is another pathway for organic pollutants and heavy metal contamination through the food web^19,20^. However, when compared to other more relevant contamination sources (*e*.*g*. prey species) marine debris is not acknowledged as the main vector for organic pollutants^21^. Despite marine debris ingestion by fish and its persistence in the environment, in the present literature^22^ they are still not considered to be a pressing issue in regards to public health. However, the understanding of ecological and oceanographic features are essential tools to evaluate the dynamics of marine debris ingestion by fish, and will likely influence the characteristics of contaminants (*e*.*g*. size, shape and colour). Which in turn, might be indicative of the availability of marine debris, distance of input sources and help clarify if fish prefer a specific set of particles.

Snooks (Centropomidae) are one of the most important living resources exploited by American coastal fisheries, with annual landings of ≈ 13,000 tons on the east side of the continents^23^. Adult snooks are usually found in the outermost portion of the estuary^24^ but also use habitats with greater structural complexity and migrate towards the inner habitats of the estuary in search of food and shelter^25^. Earlier stages are usually associated with nursery grounds in the mangrove creeks and upper estuary^24,26^. Snooks are one of the main estuarine top predators, occupying a demersal habitat, feeding mainly on fishes and macrocrustaceans^27^.

Considering the ecological importance of snooks as estuarine top predators and their economic relevance in the tropical western Atlantic, studies on the patterns of microfilament contamination in these species will serve as important indicators of potential risks to humans. The aim of this study is to investigate the spatio-temporal patterns of contamination with different sizes and colours of microfilaments (marine debris) in three important commercially exploited species in the Goiana Estuary (Brazil). The Centropomidae species *Centropomus undecimalis*, *C*. *mexicanus* and *C*. *pectinatus*, were sampled throughout their ontogeny (different life history stages) to correlate their patterns of contamination with ecological use of the estuary, and establish possible pathways of microfilaments ingestion.

## Results

Microfilaments were the major type of marine debris ingested by the snooks, representing more than 98% of the 773 particles ingested. Other types of marine debris such as hard particles (<1%), soft particles (<1%) and paint chips (<0.3%) were also found, but they not included in the analysis due to their low ingestion rates (Supplementary Fig. S1).

From a total of 529 fishes analysed, 306 (~58%) were contaminated with microfilaments. In effect, 58% of *C*. *undecimalis* (1.51 ± 0.13 particles individual^−1^; 149 individuals), 65% of *C*. *mexicanus* (1.43 ± 0.11 part. ind.^−1^; 117 individuals) and 51% of *C*. *pectinatus* (1.21 ± 0.18 part. ind.^−1^; 40 individuals) were contaminated. To evaluate the potential input sources of marine debris to the environment, the ingested microfilaments were measured and divided into six different colours (blue, purple, green, red, white and black).

### Size and colours of ingested microfilaments

**Figure 1:**
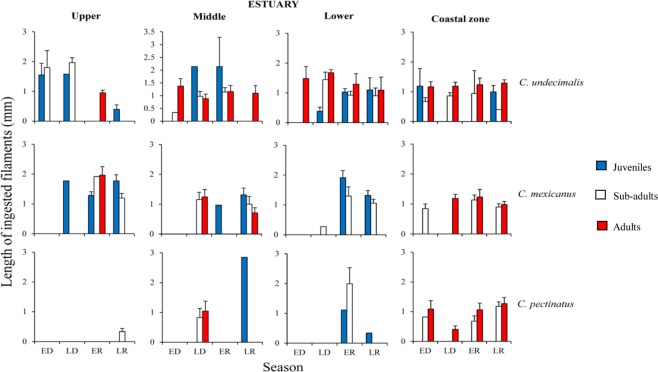
Mean (SE±) length of microfilaments ingested by the snooks, regarding different habitats (upper, middle, lower estuary and coastal zone), seasons [ED (early dry), LD (late dry), ER (early rainy) and LR (late rainy)] and ontogenetic phases.

Individuals of *C*. *undecimalis* and *C*. *mexicanus* ingested longer microfilaments in the upper estuary (1.41 ± 0.20 mm and 1.52 ± 0.09 mm, respectively) and smaller sizes in the coastal zone (1.08 ± 0.05 mm and 1 ± 0.06 mm, respectively) (Supplementary Table S1). *C*. *pectinatus* ingested longer microfilaments in the lower estuary (1.63 ± 0.44 mm), but in the coastal zone they followed the same trend as the other species (1.02 ± 0.08 mm) (Fig. 1).

Interactions among habitat *vs*. season *vs*. ontogenetic phases significantly affected the size of filaments ingested (Fig. 1; Supplementary Table S2). For *C*. *undecimalis*, the longest microfilaments were ingested by juveniles in the upper estuary, during the early dry season (1.55 ± 0.38 mm).

For *C*. *mexicanus*, the longest microfilaments were ingested in the upper estuary, during the early rainy season (adults) (1.96 ± 0.28 mm) and late rainy season (juveniles) (1.77 ± 0.20 mm). For *C*. *pectinatus*, the longest microfilaments were recorded in sub-adults in the lower estuary, during the early rainy season (1.99 ± 0.53 mm). Smaller microfilaments were commonly ingested in the upper reaches of the estuary, however, no significant differences were detected.

Fishes were more prone to be contaminated in the lower estuary and during the rainy season (Supplementary Table S1). Regardless of colour, adult snooks registered the highest rates of contamination. The majority of filaments ingested by snooks were blue (75.9%), followed by red (6.9%), green (6%), purple (5.8%), white (4.9%) and black (0.1%).

**Figure 2:**
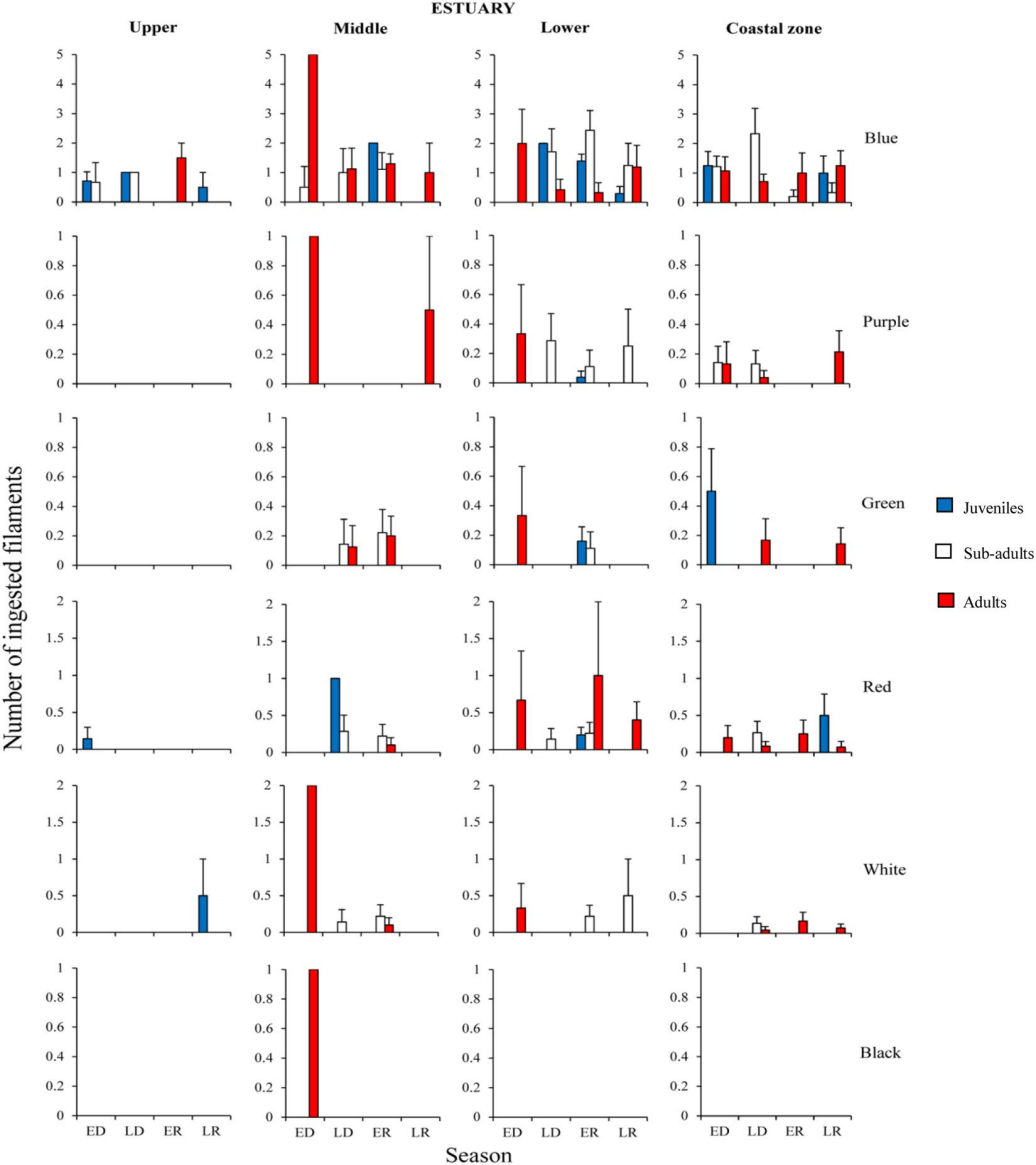
Mean (SE±) number of different colours of microfilaments ingested by the *C*. *undecimalis*, regarding different habitats (upper, middle, lower estuary and coastal zone), seasons [ED (early dry), LD (late dry), ER (early rainy) and LR (late rainy)] and ontogenetic phases.

**Figure 3:**
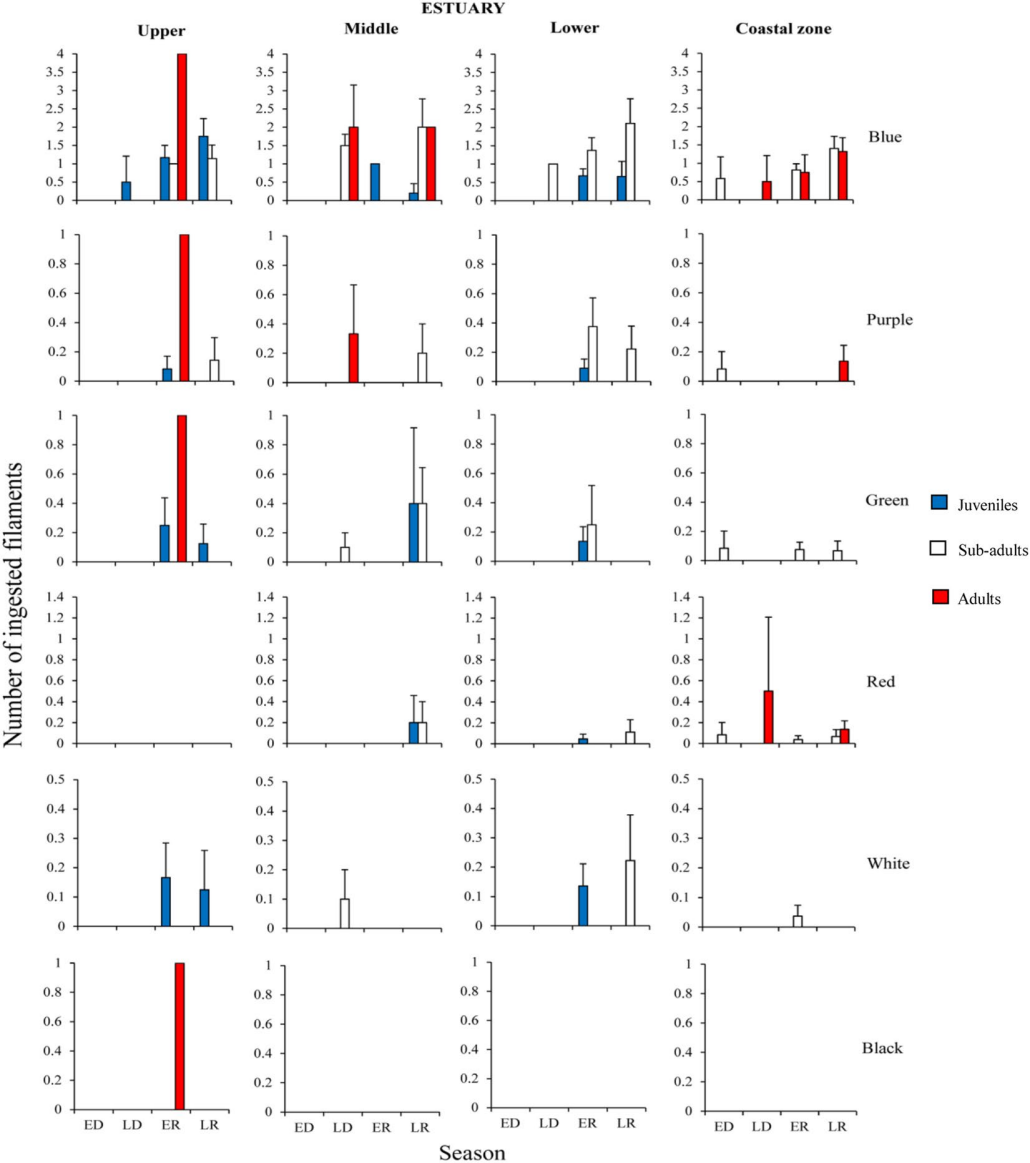
Mean (SE±) number of different colours of microfilaments ingested by the *C*. *mexicanus*, regarding different habitats upper, middle, lower estuary and coastal zone), seasons [ED (early dry), LD (late dry), ER (early rainy) and LR (late rainy)] and ontogenetic phases.

For snooks of all ontogenetic phases the highest contamination rates of blue microfilaments occurred during the rainy season in the lower estuary and coastal zone (Figs 2, 3 and S2). However, juveniles (1.4 ± 0.23 part. ind.^−1^) and sub-adults (2.44 ± 0.66 part. ind.^−1^) of *C*. *undecimalis* had the highest contaminations in the lower estuary, during the early rainy season (*p* < *0*.*01*) (Fig. 2; Supplementary Table S3).

Sub-adults of *C*. *mexicanus* were mostly contaminated during the late rainy season in the middle (2 ± 0.77 part. ind.^−1^) and lower (2.11 ± 0.67 part. ind.^−1^) estuaries (*p* < *0*.*01*) (Fig. 3; Supplementary Table S4). Sub-adults and adults of *C*. *pectinatus* were more contaminated in the coastal zone, during the late rainy season (0.94 ± 0.29 part. ind.^−1^ and 1.28 ± 0.45 part ind.^−1^, respectively) (*p* < *0*.*01*) (Supplementary Fig. S2; Table S5).

A similar pattern was detected for purple microfilaments. Higher contamination rates were observed in all species inhabiting the outermost habitats (Figs 2, 3 and S2). Sub-adult of *C*. *mexicanus* had the highest ingestion rates in the lower estuary, during the early rainy season (0.37 ± 0.19 part. ind.^−1^; *p* < *0*.*05*) (Fig. 3; Supplementary Table S4). Meanwhile, adults of *C*. *undecimalis* (Fig. 2; Supplementary Table S3) and *C*. *pectinatus* (Supplementary Fig. S2; Table S5) were most contaminated in the coastal zone (*p* < *0*.*01*), during the late rainy season (0.21 ± 0.14 part. ind.^−1^ and 0.42 ± 0.21 part. ind.^−1^, respectively).

Green microfilaments were ingested throughout the habitats used by *C*. *undecimalis* and *C*. *mexicanus* (Figs 2 and 3). The highest ingestion rates for *C*. *undecimalis* occurred in sub-adults (0.22 ± 0.15 part. ind.^−1^) and adults (0.2 ± 0.13 part. ind.^−1^) in the middle estuary during the early rainy season (*p* < 0.05). Additionally, juveniles had the highest concentrations (0.16 ± 0.09 part. ind.^−1^) in the lower estuary during the early rainy season (*p* < 0.05) (Fig. 2; Supplementary Table S3). In the coastal zone, the highest contamination of juveniles was recorded during the early dry season (0.5 ± 0.28 part. ind.^−1^), and in adults during late dry and late rainy seasons (0.16 ± 0.14 part. ind.^−1^ and 0.14 ± 0.11 part. ind.^−1^, respectively) (*p* < 0.05).

Red microfilaments ingestion peaked in adult *C*. *undecimalis* in the lower estuary, during the early rainy season (1 ± 1 part. ind.^−1^) (Figs 2, 3 and S2). Adults of *C*. *mexicanus* ingested more red microfilaments in the coastal zone, during the late dry season (0.5 ± 0.7 part. ind.^−1^) and sub-adults of *C*. *pectinatus* ingested more in the middle estuary, during the late dry season (0.5 ± 0.5 part. ind.^−1^).

The highest ingestion rates of white microfilaments were detected in sub-adults of *C*. *mexicanus* in the lower estuary, during the late rainy season (0.22 ± 0.15 part. ind.^−1^; *p* < *0*.*01*) (Fig. 3; Supplementary Table S4). Peaks of ingestion were registered for juvenile *C*. *undecimalis* in the upper estuary, during the late rainy season (0.5 ± 0.5 part. ind.^−1^), in the lower estuary for both, sub-adults of *C*. *undecimalis* during the late rainy season (0.22 ± 0.14 part. ind.^−1^) and for sub-adults of *C*. *pectinatus* during the early rainy season (0.5 ± 0.5 part. ind.^−1^) (Fig. 2 and S2; Supplementary Tables S3 and S5).

### Feeding behaviour

The diet of snooks included a wide range of prey, which were grouped into six major ecological/taxonomic groups (pelagic fishes, demersal fishes, macrocrustaceans, microcrustaceans, bristle worms and organic matter) (Supplementary Table S6).

Juveniles consumed the most variable diet, with macrocrustaceans and bristle worms being the most important (Supplementary Figs S3, S4 and S5). The highest ingestion of bristle worms and organic matter was recorded for juveniles of *C*. *mexicanus* in the lower estuary, during the early rainy season (140.8 ± 62.1 mg ind.^−1^ and 23.2 ± 14.8 mg ind.^−1^, respectively; *p* < *0*.*01*) (Supplementary Fig. S4; Table S7). In contrast to the other species, juveniles of *C*. *undecimalis* had higher intakes of both pelagic and demersal fishes in the lower estuary and coastal zone.

Sub-adults snooks exhibited a transitional feeding behaviour, preying mostly on macrocrustaceans, microcrustaceans and bristle worms in the inner sections of the estuary. In the outer sections they fed mostly on pelagic fishes, demersal fishes and macrocrustaceans. For sub-adults of *C*. *undecimalis*, the highest ingestion of pelagic fishes occurred in the coastal zone, during the late dry season (3,166.7 ± 1,395.5 mg ind.^−1^; *p* < 0.05) (Supplementary Fig. S3; Table S8). Pelagic fishes were also the main prey of sub-adult *C*. *mexicanus*, which registered the highest ingestion rates of this resource in the coastal zone, during the early rainy season (3,205.7 ± 579.2 mg ind.^−1^; *p* < *0*.*01*) (Supplementary Fig. S4; Table S7). Sub-adults of *C*. *pectinatus* had the highest ingestion rates of both macrocrustaceans and organic matter in the coastal zone, during the late rainy season (282.7 ± 104.8 mg ind.^−1^ and 17.1 ± 9.8 mg ind.^−1^, respectively; *p* < *0*.*01*) (Supplementary Fig. S5; Table S9).

Adult snooks fed mostly on pelagic fishes, demersal fishes and macrocrustaceans, with pelagic fishes being the main food resource. Adults of *C*. *undecimalis* had the highest ingestion of pelagic fishes in the coastal zone throughout the seasonal cycle (*p* < *0*.*05*) [early dry (8,328.9 ± 2,643.7 mg ind.^−1^), late dry (6,654.4 ± 2,407 mg ind.^−1^), early rainy (7,509.2 ± 4,018.9 mg ind.^−1^) and late rainy (3,936.4 ± 1,306.8 mg ind.^−1^) seasons] (Supplementary Fig. S3; Table S8). Similarly, adult *C*. *mexicanus* had the highest ingestion of pelagic fishes in the coastal zone, but only during the late rainy season (6,971.4 ± 3,272.3 mg ind.^−1^; *p* < *0*.*01*) (Supplementary Fig. S4; Table S7). Meanwhile, adults of *C*. *pectinatus* registered the highest intake of demersal fishes in the coastal zone, during the early rainy season (3,584.2 ± 3,584.2 mg ind.^−1^; *p* < *0*.*01*) (Supplementary Fig. S5; Table S9).

### Influences of environmental variability in the patterns of microfilament contamination

**Figure 4:**
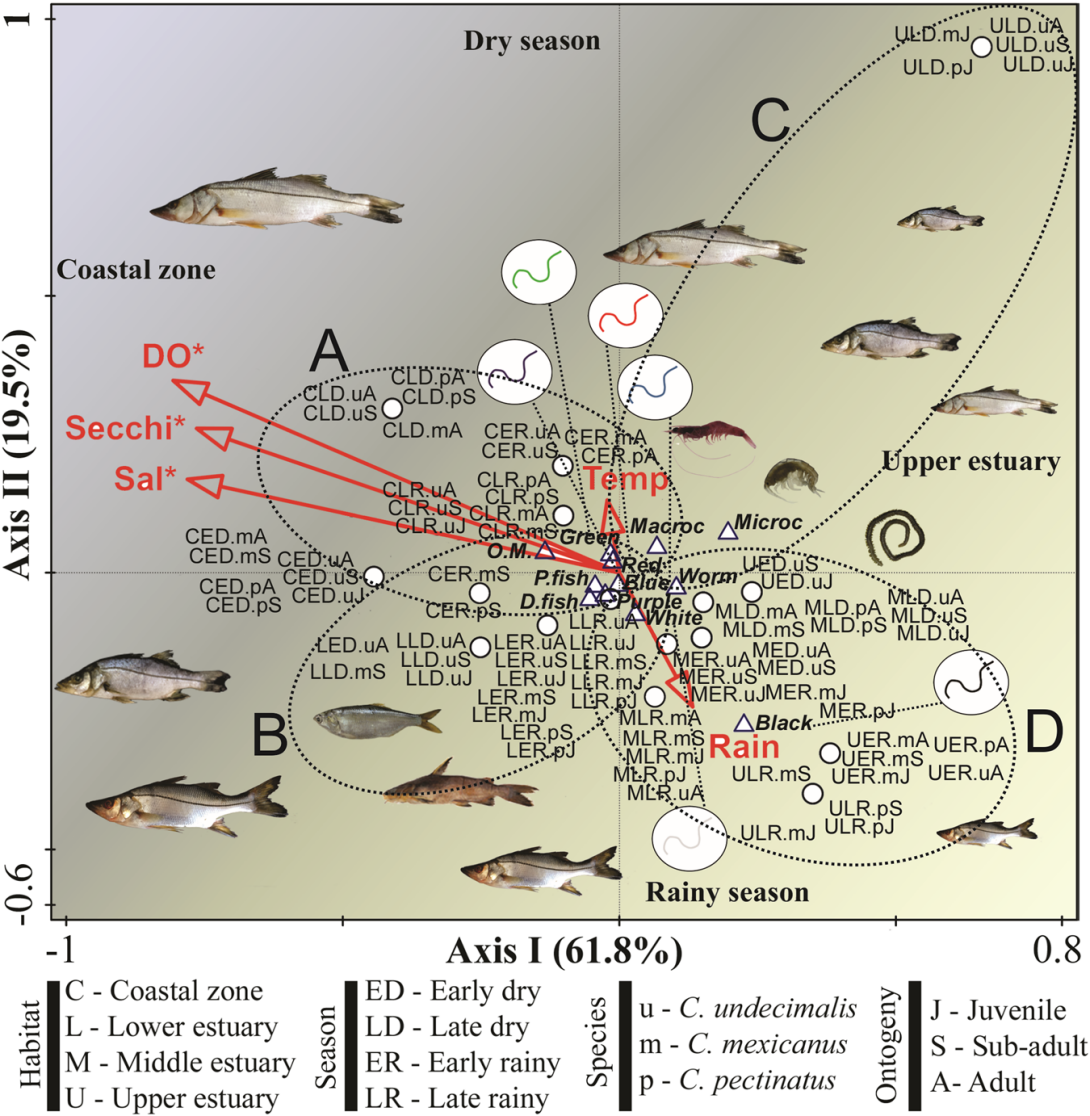
Canonical Correspondence Analysis (CCA) for the correlations among the different colours of microfilaments ingested, main food groups and environmental variables. Vectors represent the environmental variables [Sal (salinity), Secchi (Secchi depth), DO (dissolved oxygen), Temp (water temperature) and Rain (rainfall). Circles represent the interactions among the factors habitats, seasons, and ontogenetic phases of species. Triangles represent the colours of microfilaments ingested [Blue (blue microfilaments), Purple (purple microfilaments), Green (green microfilaments), Red (red microfilaments), White (white microfilaments) and Black (black microfilaments)] and the main food groups [Pfish (pelagic fishes), Dfish (demersal fishes), Macroc (macrocrustacens); Microcr (microcrustaceans); Worm (bristle worms) and O.M. (organic matter)].

The CCA was used to evaluate the relationship among the different colours of microfilaments, main food resources ingested and the environmental parameters of the ecosystem (Fig. 4). Axis I of the analysis explained 61.8% of the data variability, being negatively correlated with salinity (*p* < 0.01), dissolved oxygen (*p* < *0*.*01*) and Secchi depth (*p* < *0*.*01*) (Fig. 4). Axis I represented the salinity ecocline of the ecosystem. The positive section of this axis represented the innermost habitats (upper and middle estuaries) and the negative section the outermost habitats (lower estuary and coastal zone). The axis II explained 19.5% of the variability, being positively correlated with water temperature, salinity, dissolved oxygen and Secchi depth and negatively correlated with rainfall. Axis II described the seasonality. Its negative section represented the increased influence of river discharge in the ecosystem, which occurs during the rainy seasons. The positive section represented the increased oceanic influence that is more intense during the dry seasons.

Blue, purple, green and red microfilaments were placed near to the intersection of both axes. These were the filament colours which caused the highest contamination rates among the snooks. Additionally, they were placed slightly towards the negative section of axis I because of higher ingestions rates in the outermost habitats (Fig. 4 and Supplementary Table S10). Group A represented snooks contaminated in the coastal zone and in the lower estuary, during the dry season (Fig. 4). Whereas group B included the most contaminated fishes, mainly from the lower estuary and coastal zone, during the rainy seasons. Group C and D represented the individuals that ingested microfilaments in the habitats with prevailing riverine influence, during the dry and rainy seasons, respectively.

The food groups, pelagic and demersal fishes were positively correlated with salinity, dissolved oxygen, Secchi depth and rainfall. Despite being consumed in all habitats and seasons, most items were consumed in the lower estuary and coastal zone, especially in the rainy seasons. Moreover, the CCA plotted the pelagic fishes and demersal fishes close to blue and purple microfilaments, suggesting that snooks have similar patterns of consumption/contamination for these items.

Macrocrustaceans and organic matter were positively correlated with temperature and negatively correlated with rainfall. However, macrocrustaceans were associated with the innermost habitats and organic matter with the outermost habitats (Fig. 4 and Supplementary Table S10). White microfilaments, bristle worms and microcrustaceans were also associated with the innermost habitats, being mostly ingested in the upper and middle estuaries but also in the lower estuary, during the rainiest seasons (Fig. 4 and Supplementary Table S10).

## Discussion

Microfilaments are widely distributed in aquatic ecosystems, with reports of many contaminated^28,29^ and few non-contaminated taxa^30^. Hydrodynamic forces and distance from significant sources are the major factors influencing microfilament availability in the environment^22,31^. As a result, microfilament concentrations vary greatly among different ecosystems and even among habitats^32^. Habitats along environmental gradients are susceptible to seasonal variations that alter microfilament availability^33^. Microfilament availability in the environment and contamination levels are directly linked to the patterns of habitat use by fishes^14,34,35^. This increases the likelihood of microfilament transfer throughout the trophic chain and may ultimately lead to human contamination^36^. Therefore, to understand patterns of microfilament contamination it is important to understand the ecological behaviour of fishes for both environmental conservation and future food safety.

Ontogenetic changes through the life cycles of snook species play an important role in their ecological behaviour, leading to shifts in habitat use and feeding ecology^37^, which could also affect the dynamics of microfilament ingestion. The feeding behaviour of snooks strongly reflects the availability of microfilaments ingested, regardless of colour and size. The highest contamination rates were recorded, when the feeding behaviour of snooks switched to concentrating on prey of higher trophic levels (*e*.*g*. pelagic and demersal fishes).

Juveniles of *C*. *undecimalis* and *C*. *mexicanus* were classified as opportunist predators and juveniles of *C*. *pectinatus* as zoobenthivorous^38^. Sub-adult snooks were classified as opportunistic predators and adults as piscivorous. The highest ingestion rates of microfilaments were registered in adults of *C*. *undecimalis*, followed by sub-adults of *C*. *undecimalis* and adults of *C*. *mexicanus* and *C*. *pectinatus*.

Contamination with microfilaments is, in this case, a result of the trophic transfer and, as a result, species of higher trophic levels were more contaminated^14,39^. Trophic transfer of microfilaments occurs when a contaminated prey is ingested and during the digestive process the microfilaments that were within the digestive tract of the prey are transferred to the predator^40^. Evidence of this process has been observed in other estuarine fishes, such as Sciaenidae, acoupa weakfish (*Cynoscion acoupa*) and little croaker (*Stellifer stellifer*). In both cases, the adult phase fed mostly on fishes and had the highest levels of contamination with microfilaments^41,42^. Trophic transfer was also reported for the Brazilian mojarra (*Eugerres brasilianus*) and the flagfin mojarra (*Eucinostomus melanopterus*)^43^.

**Figure 5:**
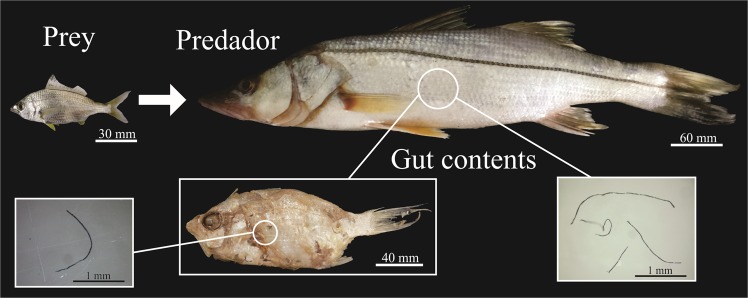
Evidence of trophic transfer observed between the predator (*C*. *undecimalis*) and prey (*Eucinostomus melanopterus*). Both individuals were contaminated by microfilaments.

Further evidence that indicates the contribution of trophic transfer to the contamination rates of microfilaments was evinced through the prey ingested by snooks. Some food items, at an early stage of digestion, were retrieved from the guts of snooks. Those items had their digestive tract inspected in search of microfilaments. From the 41 ingested items analysed in these conditions, 58% were contaminated with microfilaments (Fig. 5).

Moreover, proximity to the input source appears to be a major aspect influencing the different colours of microfilaments ingested. However, the predominance of contamination with blue microfilaments, is so vast that their ingestion is usually greater than the total of the other colours of microfilaments^14,44^. The high ingestion rate of microfilaments, in comparison to low concentrations in the water column^32^ and sediment^9^ implies that there is an active selection and/or pre-concentration process operating, either through selective consumption by younger snooks or through bioaccumulation through the food web.

Goiana City is a major upstream source of contaminants, and the artisanal fishery fleet is close to the mouth of the estuary^45^. Urban effluents^46,47^ and the fishing activity (mainly blue microfilaments)^48,49^ are then indicated as the main sources of microfilaments in the estuary. Due to their widespread intake by snooks, blue microfilaments are likely to have both riverine and estuarine origins.

Blue microfilaments were ingested throughout the length of the estuary, during the entire seasonal cycle, by all Centropomidae species. The highest ingestion rates occurred during the rainy seasons in the lower estuary, and its surroundings habitats (middle estuary and coastal zone). This is likely a consequence of higher microfilaments availability, coinciding with the peak in the fishing activity in the estuary^7^. Indeed, fishes are regularly reported with microfilament contamination that originates from fishing gear^44,50,51^.

The highest contamination rates of purple microfilaments occurred in the lower estuary and coastal zone by older snooks. It is likely a result of increased weathering of marine debris into microfilaments. In turn, microfilaments may alter their colour, size and/or physical characteristics (*i*.*e*. weathered purple microfilaments may resemble blue microfilaments). The reason for the highest ingestion rates of this colour being recorded in the sub-adult and adults of all species is their ecological behaviour. These ontogenetic phases had higher densities in these habitats and fed mostly on prey of higher trophic levels, thus increasing the chances of trophic transfer.

The ingestion of green microfilaments occurred differently among the species. *C*. *undecimalis* ingested green microfilaments throughout the seasonal cycle and its ontogeny, except in the upper estuary. On the other hand, *C*. *mexicanus* was mostly contaminated in the upper estuary, specifically during the early rainy season. Meanwhile, *C*. *pectinatus* ingested very few green microfilaments.

Red microfilaments were mostly ingested in the lower estuary, especially in the coastal zone. Higher contamination rates were detected in the lower and coastal habitats, it is, therefore, likely that the main input source for this colour of filament is from coastal waters, which may be carried into the estuary by waves and tides. Snooks were also contaminated with red microfilaments in the upper estuary, but to a lesser degree. Indeed, the highest ingestion rates were recorded in the middle and lower portions of the estuary and occurred when the saline intrusion was dislocated to these habitats, during the dry and rainy seasons, respectively^41^. The saline intrusion works as a barrier, preventing the passage of contaminants carried by the oceanic waters towards the upper reaches of the estuary^52^. This results in reduced availability and contamination rates with microfilaments of oceanic origin, such as red microfilaments.

Microfilaments carried by the river flow tend to become trapped within the estuarine/oceanic boundary, due to the barrier effect caused by the confluence of riverine and oceanic waters^33^. The ingestion of white microfilaments was strongly associated with the rainy season in all habitats. This is indicative of an origin related in river discharge, with sewage being the likely main input source of white microfilaments.

A number of studies have reported the possibility that macro marine debris (>5 mm) and even micro marine debris are intentionally ingested by marine biota, due to the resemblance of debris to natural prey^53,54^. Taking into account that white microfilaments are more similar in colour and size to microcrustaceans (a group formed mostly of zooplankton), white microfilaments would be expected to be preferentially ingested by juveniles and sub-adults in the upper and middle estuaries, where microcrustaceans form a large proportion of their diet. However, no associations were observed between microcrustaceans and microfilaments, suggesting that microfilament ingestion associated with microcrustacean prey is not a relevant pathway for contamination of snooks.

The largest difference in the average size of microfilaments ingested by snooks was correlated with the habitat in which they were ingested (availability). In habitats with greater riverine influence, fishes ingested longer filaments. Whereas, in the outermost habitats with greater oceanic influence, fishes ingested shorter filaments. These patterns are likely the result of the proximity of the contaminant and their input source^14^. Rivers receive great amounts of debris mostly from cities located along their margins, which are important pathways for the transportation of debris from land-based sources into the ocean^55^. Hydrodynamics are an important erosive agent to marine debris, which breaks down into smaller particles^8^. Thereby, the lower estuary and the coastal zone are the habitats most likely to have smaller particles due to intense turbulence caused by the convergence of riverine discharge and tidal flow, and the consequent breakdown of larger particles. This is reflected in the smaller sizes of microfilaments ingested by fishes in the lower phases of the estuary.

Additionally, another trend for the ingestion of longer filaments was observed. Juveniles of *C*. *mexicanus* and sub-adults of *C*. *pectinatus* also ingested longer filaments in the lower estuary, but only during the early rainy season. This occurred concomitantly with the peak of the fishery activity in the Goiana Estuary, which is responsible for an intense input of microfilaments into the lower estuary^7^.

Supposedly, the larger the fish, the greater would be the chances to ingest bigger microfilaments^43^. Additionally, macrofilaments are more readily detected by fishes (due to their greater dimensions), but this category was rarely ingested. No evidence of selective ingestion of microfilaments, either in size or colour, were observed among the ontogenetic phases of snooks, suggesting that direct ingestion of marine debris from the water column by predatory fishes such as snooks is not relevant.

**Figure 6:**
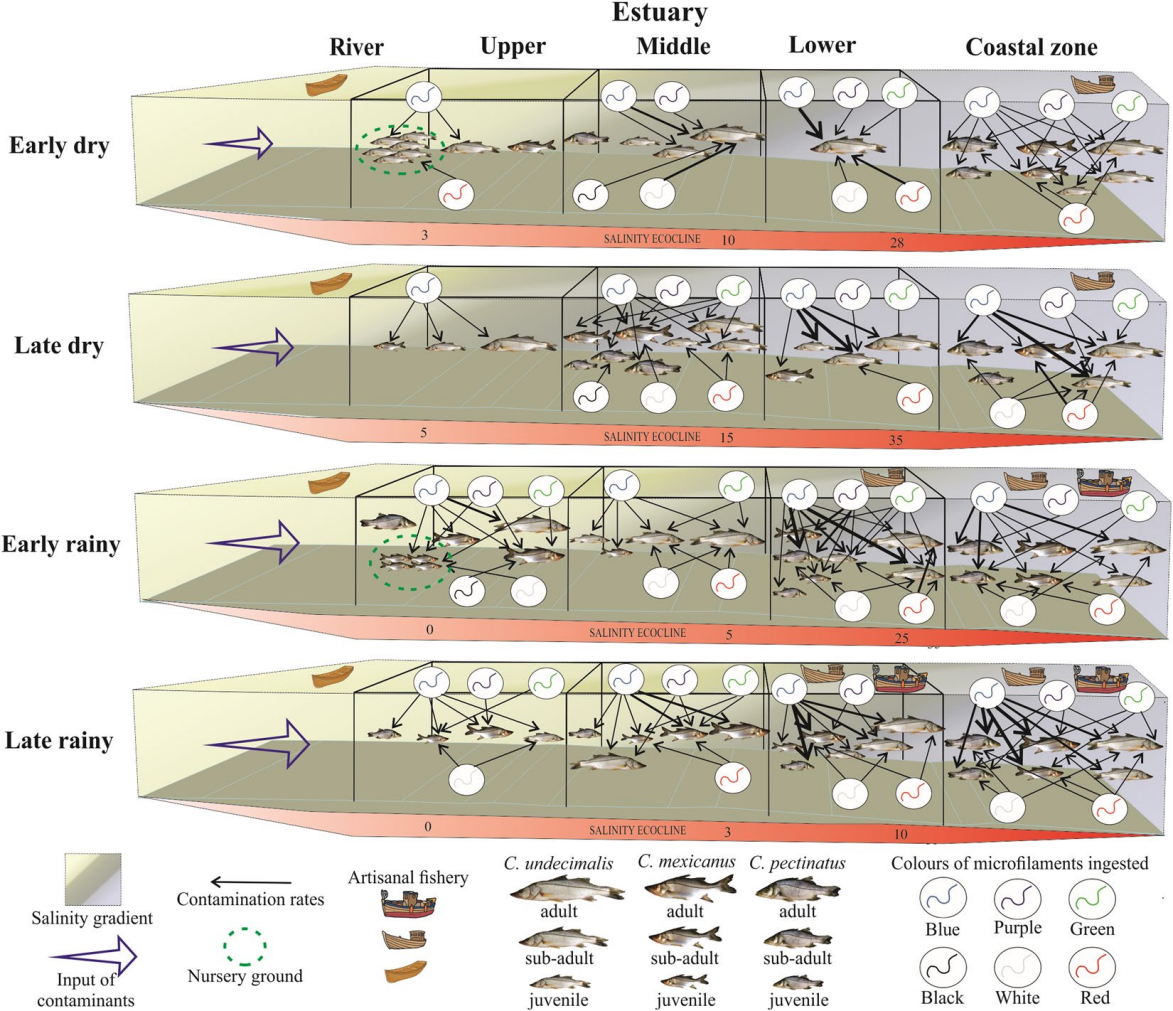
Conceptual model for the ingestion rates of different colours of microfilaments by snooks, regarding different habitats, seasons and ontogenetic phases.

Evidence suggests that trophic transfer is the most important influence on the total quantity of microfilaments in Centropomidae species, with different contamination rates recorded through ontogeny. Moreover, the peaks of ingestion of microfilaments of different colours, seems to be associated with the proximity to their input sources, being closely correlated with the seasonal variability of the salinity structure in the estuary. Multiple factors were noteworthy contributors of the contamination patterns, including ecological behaviour of each species, seasonality, river discharge, hydrodynamics of the estuarine boundary and local fishery fleet (Fig. 6). The size of filaments ingested by the fishes is clearly associated with the salinity ecocline. The ingestion of different colours of microfilaments is likely a result of their availability in the environment to prey. No evidence indicated fish preferences for specific colours or sizes of microfilaments.

Studies on the consequences of marine debris ingestion for biota are still incomplete, especially regarding multilevel trophic processes. Effects on animals from chemical additives that are potential endocrine disruptors^56^; their relevance as vectors for organic pollutants adsorbed from the environment^21,57^ and influence on the behaviour patterns of fish^40,58^ are becoming more common in the literature. However, the trophic transfer of marine debris through the food web^14,36,39^ still poses a great concern that extends to human health, due to the consumption of contaminated seafood^17^. Little information is available on plastic effects on human health^17^, but a recent survey conducted on mice, reported accumulation of particles in vital organs, impairing molecular functions^59^. Human contamination with marine debris (including the microfilaments dealt with here), is probably related to length and intensity of exposure to contaminated food, including fish. That is, daily, weekly, yearly and life-long patterns of fish consumption. As for other pollutants, such as mercury, if risk arises, a choice might have to be made about which fish to consume based on its trophic position and age. How much and how often fish is consumed can be managed in order to control pollutants intake. In the case of marine debris, it is still uncertain if such strategies will be sufficient to reduce human exposure, and more trophic web-based information is needed to clarify that point.

Considering the ongoing magnitude of the marine debris problem, its potential hazards for wildlife and human beings we recommend assessments of marine debris ingestion for key species within regional food webs, as well as aquaculture products. Many species have been found to be contaminated, and the inventory only grows on a daily basis. The current study suggests that marine debris can enter food webs at different levels, through different routes, through different habitats and life phases. Juvenile snooks ingested microfilaments in their diets (other fish and invertebrates), starting at their nursery grounds, and likely have to deal with these loads, and further contamination, through their entire life history, eventually passing it on to their predators.

Future studies should, therefore, focus on methods that allow comparisons among studies of different ecosystems and taxa, developing sampling and laboratory protocols to improve identification of marine debris and avoid contamination biases. These include, among others, sample designs encompassing trophic, spatial and temporal variability, including an appropriate number of replicates. Despite procedural blanks being an important step to account for any contamination during collection and processing of field samples^4^, the effects of airborne contamination is likely to be negligible in surveys of fish stomach contents, particularly in studies that implement robust laboratory protocols, aligned with substantial sample sizes designed to investigate the ecological patterns of ingestion of snook species between different areas, seasons and ontogenetic phases. The development of statistical approaches, such as aquatic community modelling, will advance our understanding of how contamination by microfilaments and other pollutants (*e*.*g*. heavy metals)^60^ are correlated with trophic level, life history and the salinity ecocline, as well as other environmental gradients.

## Methods

The Goiana Estuary is located in the western tropical Atlantic Ocean. Fishes were captured from 2005 to 2015, from different habitats within the estuary (upper, middle, lower estuary, and coastal zone) and seasons (early dry, late dry, early rainy and late rainy seasons)^14,45^ (Supplementary Figs S6 and S7). Fishes samples were taken following all ethical requirements and licenced by the Environment Ministry of Brazil (SISBIO permit number: 11050).

Prior to commencing fish sampling, environmental parameters (salinity, temperature, dissolved oxygen and Secchi depth) of bottom waters were recorded and rainfall data were compiled from a local weather station. After capture, all specimens were immediately frozen and transported to the laboratory. Three species of Centropomidae were used in this study: *C*. *undecimalis*, *C*. *mexicanus* and *C*. *pectinatus*. Individuals were divided into three ontogenetic phases (juveniles, sub-adults and adults) to evaluate the contamination patterns throughout their life cycle (Supplementary Table S11).

In the laboratory, precautionary measures were taken to avoid airborne and inter-sampling contamination. To avoid airborne and inter-sampling contamination the working station and all equipment used in the evisceration were cleaned with distilled water and absolute ethanol, prior to the procedures for the identification of digestive tracts contents^61,62^. Then, tweezers, scissors, scalpels and Petri dishes were also oven dried and double checked for contamination before the next use^14,44^. Procedural blanks were not made. However, a robust sample design was applied in the study (Supplementary Fig. S7) encompassing different estuarine areas, seasons and ontogenetic phases, which included a great number of individuals of three species of snooks (n = 529). Additionally, 100% cotton lab coats and latex disposable gloves were used during all procedures^61,62^.

Fishes were then eviscerated and their digestive tracts (stomach and intestine) were removed. Their gut contents were analysed in glass covered Petri dishes (to allow identification through the lid and avoid airborne contamination) using a stereomicroscope with a digital camera attached. Items suspected of being marine debris were visually identified, separated into covered Petri dishes and oven dried in 70 °C for 48 h^63,64^. Petri dishes were kept closed during the entire identification process, with the exception of when the items were transferred to another Petri dish to be oven dried and after the confirmation for storage in the database.

Withered items were considered non-synthetic organic matter and were discarded^62^. Those items that did not changed their shape (not shrivel due to water loss and had a homogeneous thickness), physical consistency (being not easily broken or fragmented) and visual features (colour or brightness), were identified as marine debris and classified according to length, type (hard debris, soft debris, rubber crumbs, paint chips or microfilaments) and colours (blue, purple, red, green, black and white)^63^. Despite this method being a good procedure for the identification and further exclusion of non-synthetic materials from the sample, it is not a useful tool to identify the polymer the plastic debris^15,33,62^. Then, marine debris were counted, weighed (±0.0001 g), photographed and measured using the image analysis package AxioVision LE. Contaminants larger than 5 mm were not included in the study. Food items ingested by each species were counted, weighted, and categorised into six food groups (pelagic fishes, demersal fishes, macrocrustaceans, microcrustaceans, bristle worms and organic matter), according to ecological and taxonomic criteria (Supplementary Table S6).

Three-way analysis of variance was used to identify significant differences in the lengths of microfilaments ingested, the number of each different coloured microfilament and the weight of each category of food item, according to the factors: habitat, season, ontogenetic phase, and their interactions. All data were Box-Cox transformed^65^ and the ANOVA assumptions were tested. In addition, a Canonical Correspondence Analysis (CCA) was performed to investigate ecological correlations between environmental data and both the colour of microfilaments and food groups ingested by snooks [dependent variables as values of *I*_*RI*_ (Index of relative importance)^66,67^. Significant differences were accepted when α < 0.05. For details, see supplementary material.

## Supplementary information

Supplementary material
