# Supplementary material for: Dynamics of Marine Debris Ingestion by Profitable Fishes Along The Estuarine Ecocline
Source: Sci Rep. 2019 Sep 18;9:13514. doi: 10.1038/s41598-019-49992-3 (PMC6751158; doi:10.1038/s41598-019-49992-3)
Supplement: Supplementary file 1 — Supplementary material [file 41598_2019_49992_MOESM1_ESM.pdf]

SUPPLEMENTARY MATERIAL

DYNAMICS OF MARINE DEBRIS INGESTION BY PROFITABLE FISHES ALONG THE ESTUARINE ECOCLINE

Guilherme V. B. Ferreira[1], Mario Barletta[1]*, André R. A. Lima[1], Simon A. Morley[2], Monica F. Costa[1]

[1] Laboratory of Ecology and Management of Coastal and Estuarine Ecosystems, Department of Oceanography, Federal University of Pernambuco (UFPE).
Av. Arquitetura S/N, Cidade Universitária, Recife, Pernambuco CEP: 50740-550, Brazil
[2] British Antarctic Survey, Natural Environment Research Council, High Cross, Madingley Road, Cambridge, CB30ET, UK
*Corresponding author: tel: +5581994674878, +558121267223; e-mail: barletta@ufpe.br

METHODS

Study Area

The Goiana Estuary is located in the Western Tropical Atlantic Ocean, the annual water temperature is above 26 ºC, with minor fluctuations (max. 31 ºC). The estuary was spatially divided into four habitats: upper, middle, lower estuary, and coastal zone (Supplementary Fig. S6). The ecosystem seasonality is mainly influenced by the variability in rainfall, characterizing four seasons: early dry (Sep-Nov), late dry (Dec-Feb), early rainy (Mar-May) and late rainy (Jun-Aug). Rainfall is also responsible for the seasonal pulses in river flow and water quality shifts [1].

Along the estuary, there are several activities that produce solid wastes that are inadequately disposed, and are potential sources of marine debris pollution across a wide range of size fractions that also include microplastics (< 5mm). Aproximatey five kilometres upstream of the estuary, in the Goiana River, there is a city of almost 80,000 habitants, where untreated sewage effluents and urban runoff go straight into the river that drains into the estuary. Sugarcane plantations extend along the margins of the entire Goiana River towards the lower estuary, and are responsible for the input of fertilizers and pesticides into the estuary. Moreover, in the upper estuary, there are also activities such as subsistence fishery and dredging of the main channel for sand mining. In the middle estuary, the main pollution sources are the sugarcane plantations, artisanal fishery,

untreated sewage from a small fishing community and deforestation of the mangrove forest [1].

At the lower estuary and coastal zone, the main pollution sources are the fishery fleet and unplanned urbanization, where no basic sanitation is in place. Municipal services are precarious and wastes are inefficiently collected. These two habitats are under greater anthropogenic pressure. One of the main threats are the contaminants carried by the river, which are introduced along its flow, and the contaminants transported from the oceans by waves and the tidal action. Other important sources of pollutants are the disposal of effluents from a shrimp farm and a cement industry, both located in the lower estuary, and the sewage disposal from the fishing villages, located in both margins of the river mouth. However, the most relevant potential source of pollutants is likely the fishing activity, which is focused on these habitats, due to the important landing of commercial species [2].

Sampling design

Fishes were captured in the main channel of the Goiana Estuary from 2005 to 2015 encompassing different habitats and seasons (early dry, late dry, early rainy and late rainy). The upper, middle and lower portions of the estuary were sampled monthly (six replicates), using an otter trawl (forest green multifilament nylon net) between December 2005 and November 2006 (Supplementary Fig. S6) [3]. Additionally, monthly samplings (six replicates) were conducted in these same habitats, during the late dry and late rainy seasons, between December 2006 and August 2009. The mangrove creeks, located in the lower estuary, were sampled using a fyke net (forest green multifilament nylon net) (three replicates) between April and May 2008 (Supplementary Fig. S6). The net was fixed at the entrance of the creeks during high tide and then fishes were collected during the subsequent low tide. Additionally, specimens from the coastal zone adjacent to the estuary were captured monthly by the artisanal fishing fleet from 2013 to 2015 (Supplementary Fig. S6). After capture, all specimens were immediately frozen and transported to the laboratory.

Prior to fish samplings, environmental parameters were registered from bottom and surface waters [salinity (Salinometer WTW LF 197); temperature and dissolved oxygen (Oximeter WTW oxi 340); and Secchi depth]. Rainfall data were compiled from the local weather station (Supplementary Fig. S6).

Laboratory procedures

Three species of Centropomidae were used in this study: *C. undecimalis* (Common snook), *C. mexicanus* (Largescale fat snook) and *C. pectinatus* (Tarpon snook). Individuals were divided into three ontogenetic phases (juveniles, sub-adults and adults) to evaluate the diet shifts and possible contamination with microdebris through their life cycle (Supplementary Table **S**11) [4].

In the laboratory, fishes were eviscerated and the digestive tracts (stomach and intestine) were removed, their contents were analysed in a covered Petri dish using a stereomicroscope (Zeiss, Stemi 200) with a digital camera attached (Canon Powershot G10). Food items (actual prey) were visually sorted and identified to the lowest possible taxonomic level [4,5]. Then, food items were counted, weighted and categorised into six groups (pelagic fishes, demersal fishes, macrocrustaceans, microcrustaceans, bristle worms and organic matter) (Supplementary Table S6). These groups take into account ecological and taxonomic criteria. Thus, different prey within the same group has a similar foraging behaviour and each group represents a specific role within the community.

Statistical analysis

To identify significant differences in length of microfilaments ingested, number of colours, and weight of grouped food items, a three-way analysis of variance was applied for the factors: habitats, seasons and ontogenetic phases, and their interactions. Previously, to meet the ANOVA assumptions, all data were Box-Cox transformed [6] and tested for homogeneity of variances, using the Levene test, and the goodness of fit to a normal distribution, was tested using the Kolmogorov-Smirnov test[7]. Whenever significant differences were detected in the ANOVA, the sources of variance were identified using the Bonferroni *post hoc* test [8].

To investigate the ecological correlations, a Canonical Correspondence Analysis (CCA) was performed using CANOCO 5 software. This analysis investigates the influence of environmental data (independent variables) on the different colours of microfilaments and food groups ingested by Centropomidae species (dependent variables) [9]. The dependent variables were evaluated as values of I_{RI} (Index of relative importance) [10]. A triplot was produced displaying the dependent variables as geometric shapes and the independent variables as vectors. A Monte-Carlo permutation (100 permutes) was used to determinate which environmental variables significantly affected

the dependent data [9]. All statistical analysis were conducted using a 0.05 level of significance.

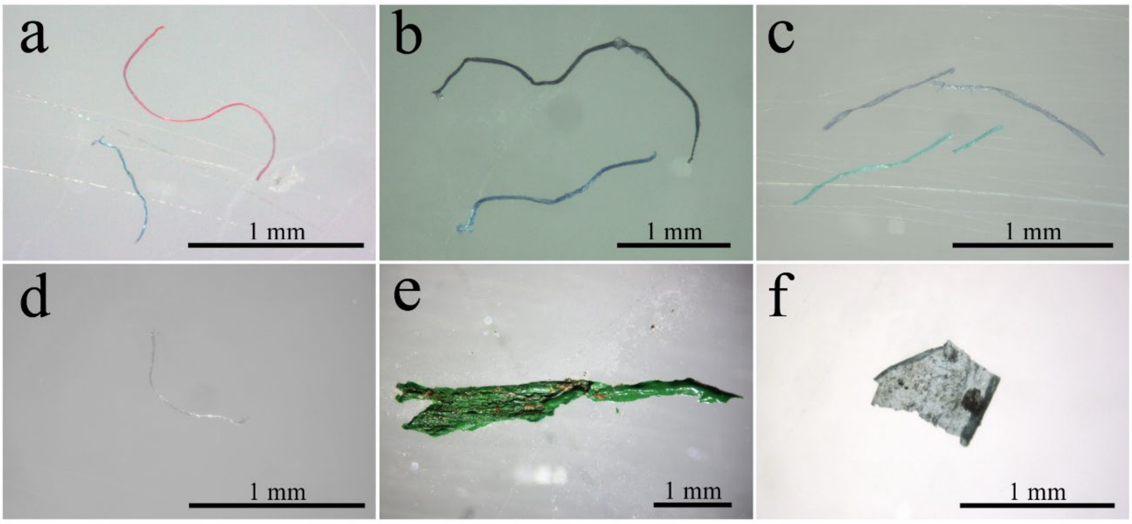

Supplementary Figure 1. Examples of microfilaments observed in the digestive tract of snooks. (a) Blue and red filaments, (b) black and blue filaments, (c) green and blue filaments, (d) white filaments, (e) green paint chips and (f) a grey soft particle.

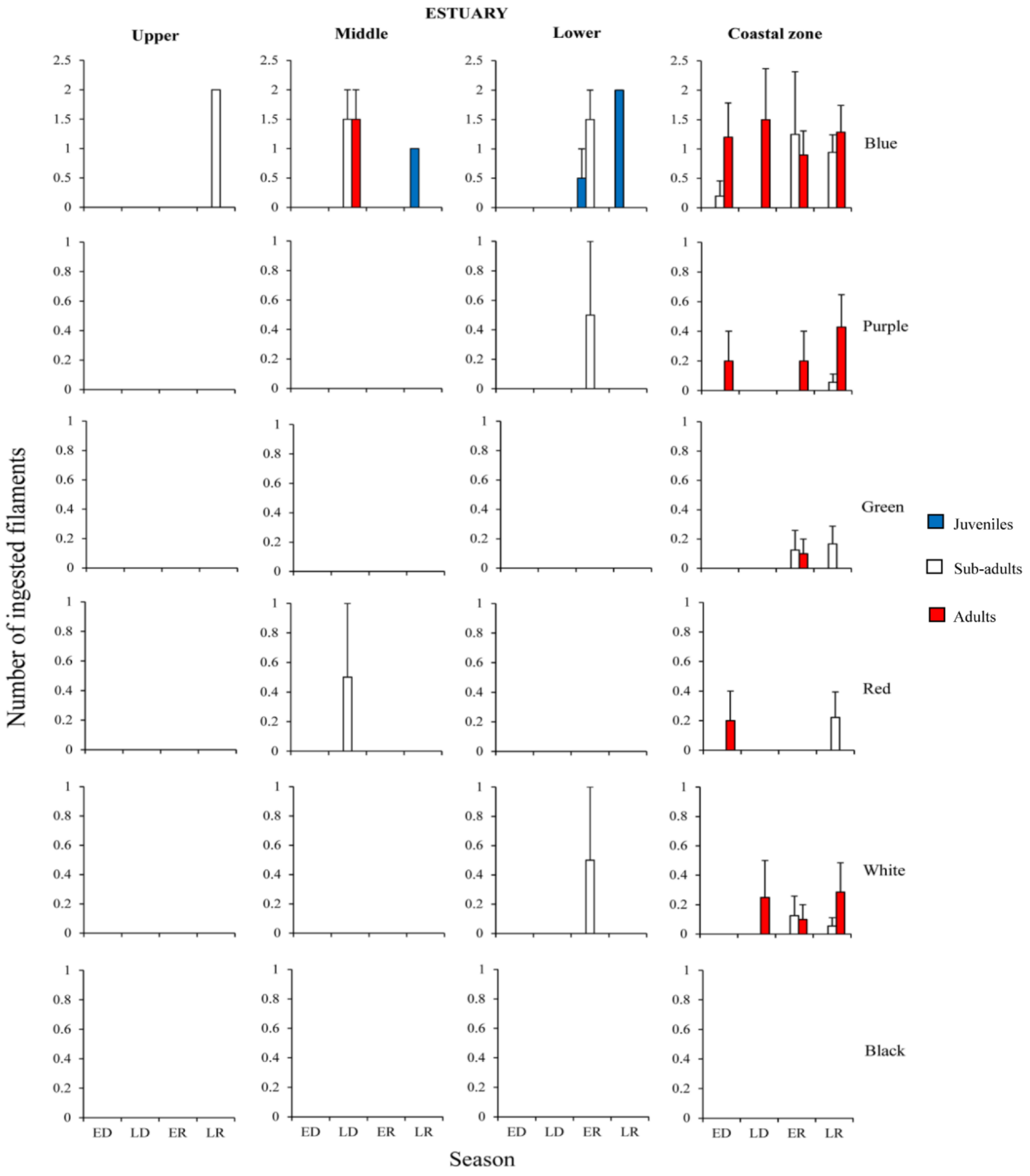

Supplementary Figure 2. Mean (SE±) number of different colours of filaments ingested by the *C. pectinatus*, regarding different habitats (upper, middle, lower estuary and coastal zone), seasons [ED (early dry), LD (late dry), ER (early rainy) and LR (late rainy)] and ontogenetic phases.

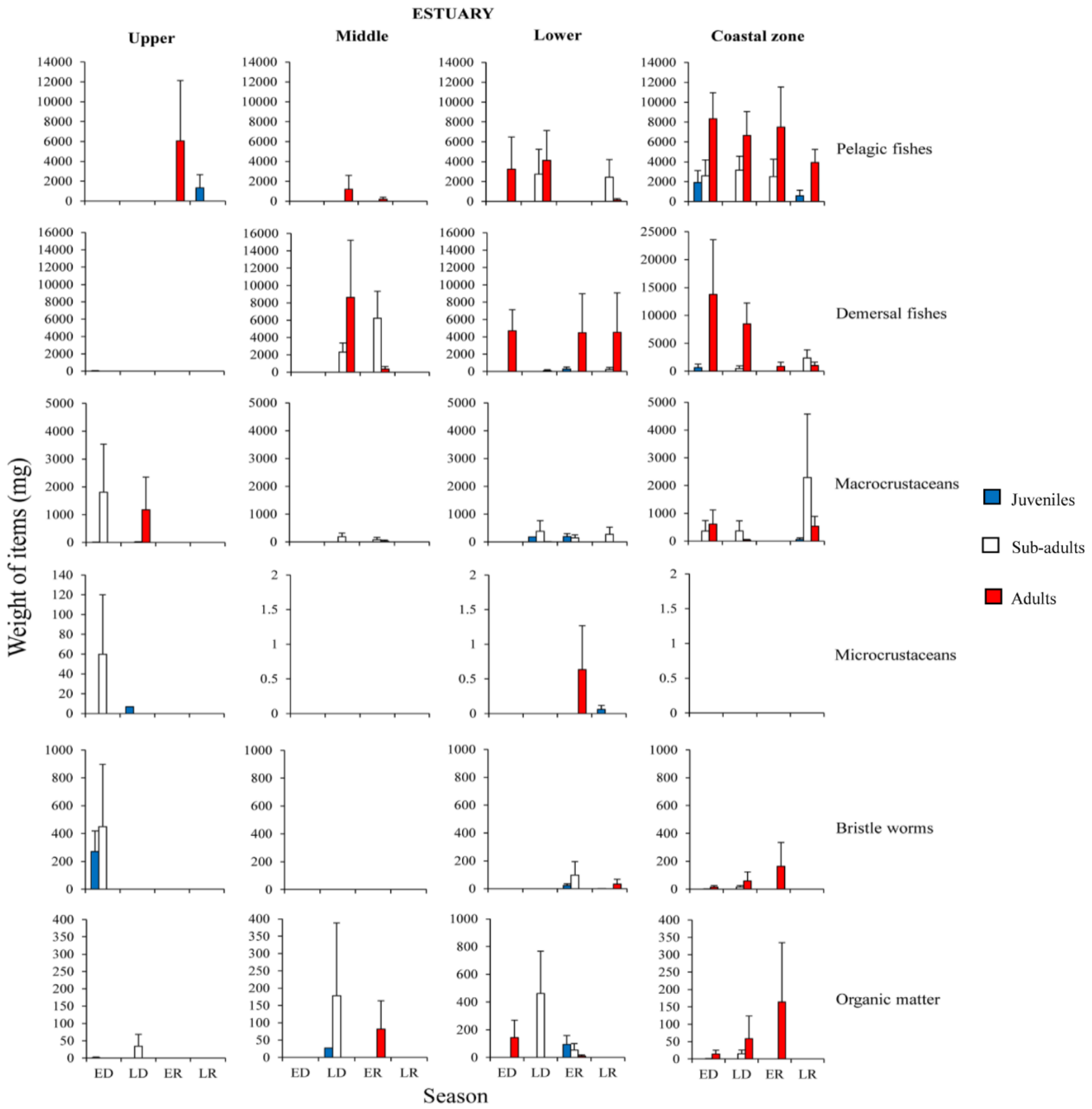

Supplementary Figure 3. Mean (SE±) weight of food groups of *C. undecimalis*, from different habitats (upper, middle, lower estuary and coastal zone), seasons [ED (early dry), LD (late dry), ER (early rainy) and LR (late rainy)] and ontogenetic phases.

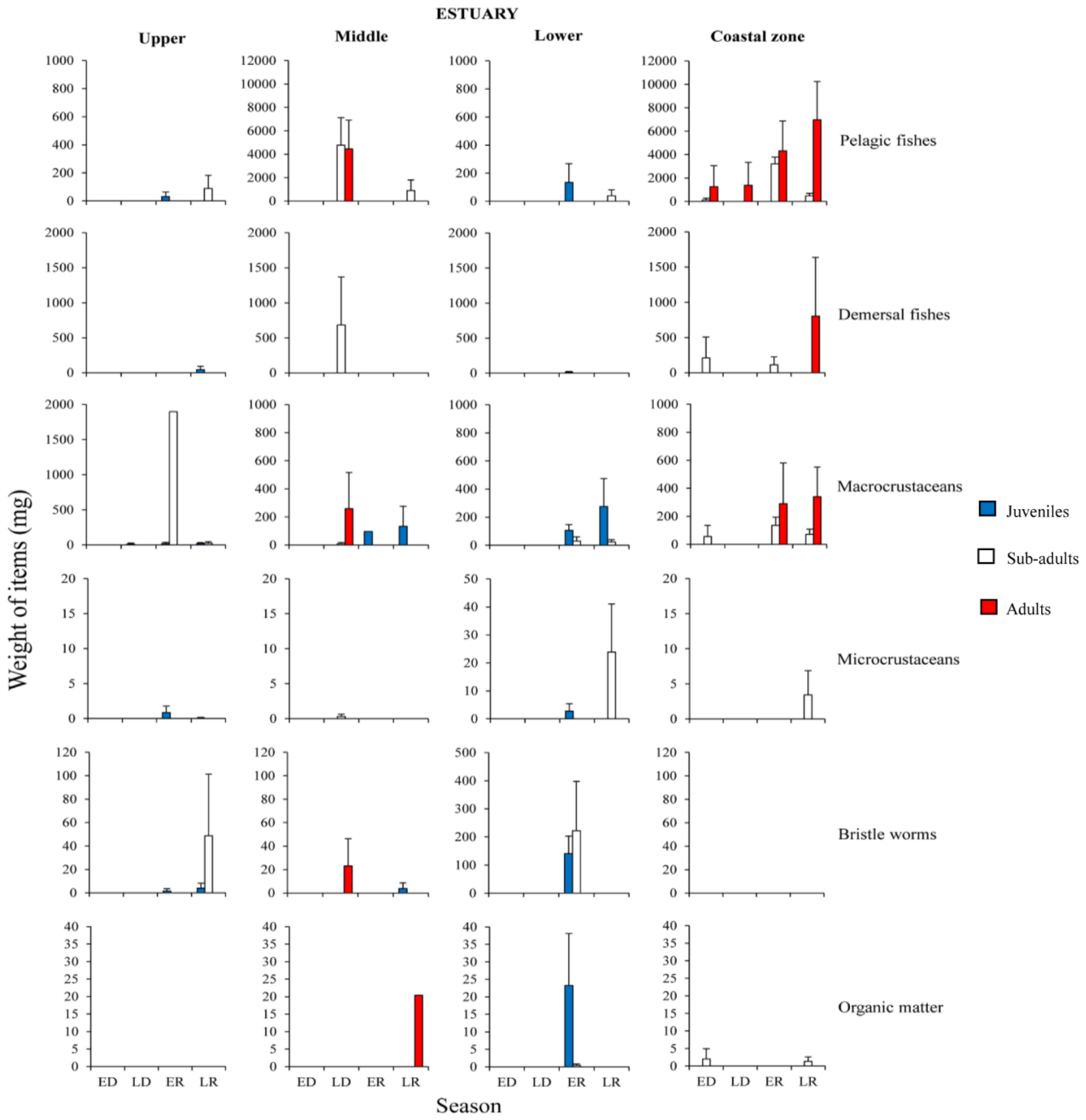

Supplementary Figure 4. Mean (SE±) weight of food groups of *C. mexicanus*, from different habitats (upper, middle, lower estuary and coastal zone), seasons [ED (early dry), LD (late dry), ER (early rainy) and LR (late rainy)] and ontogenetic phases.

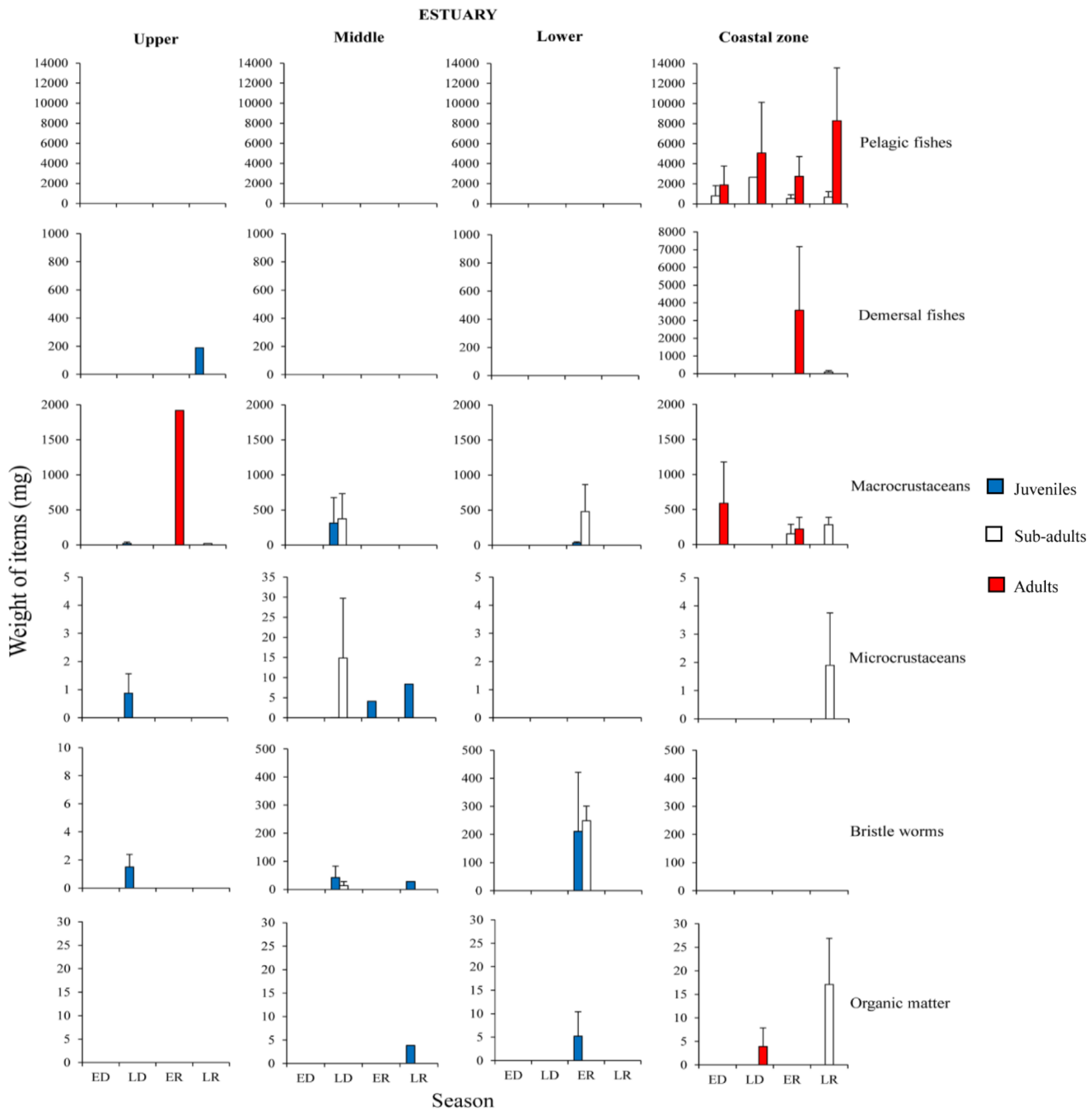

Supplementary Figure 5. Mean (SE±) weight of food groups of *C. pectinatus*, from different habitats (upper, middle, lower estuary and coastal zone), seasons [ED (early dry), LD (late dry), ER (early rainy) and LR (late rainy)] and ontogenetic phases.

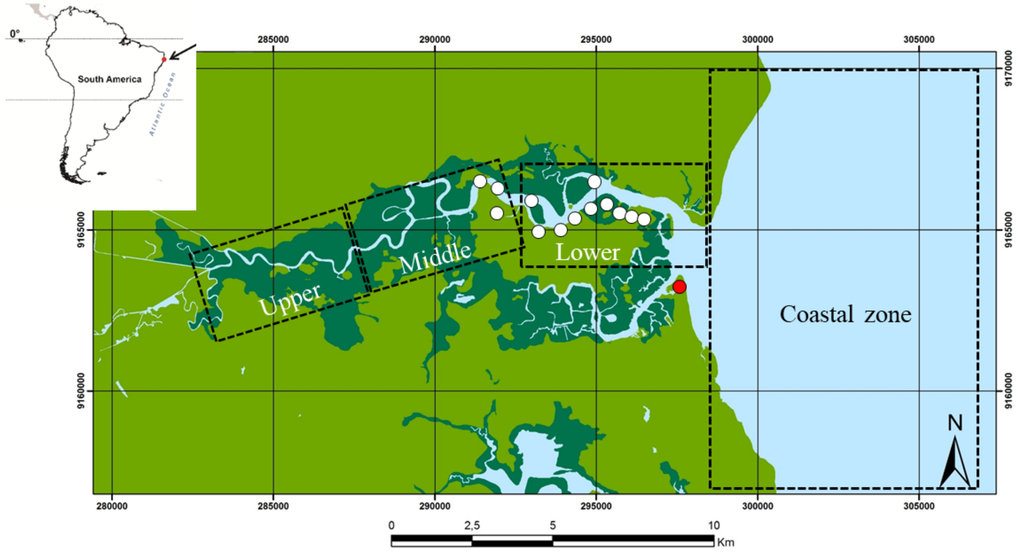

Supplementary Figure 6. Studied habitats in the Goiana Estuary: upper, middle, lower portions of the estuary, coastal zone, and (white circles) mangrove creeks. Local weather station (red circle).

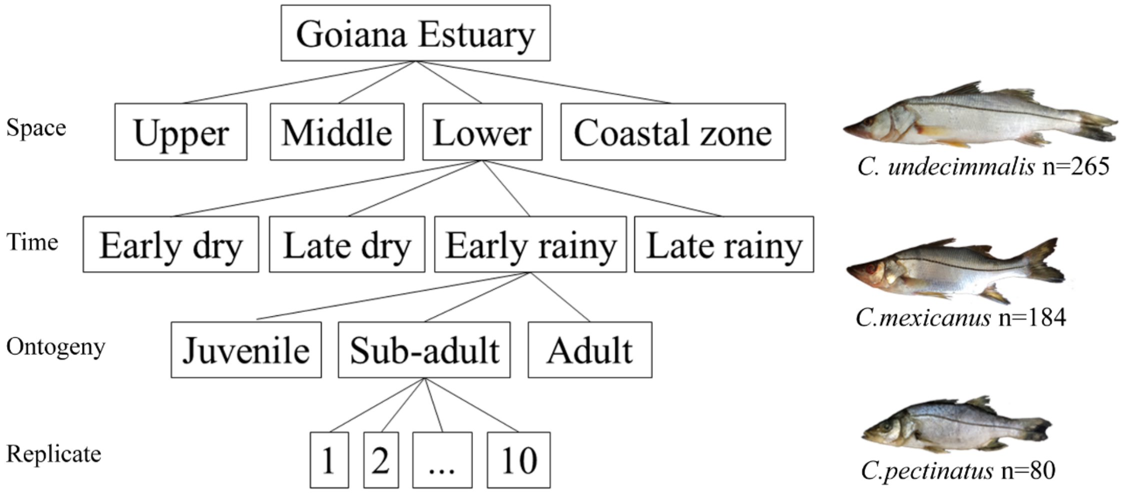

Supplementary Figure 7. Sampling design applied on the study of marine debris ingestion by Centropomidae species, according to habitat, season and ontogenetic variability.

Supplementary Table 1. Mean (SE±) number, percentage and size of microfilaments ingested by snooks, according to the factors (habitat, season, and ontogenetic phase), regardless of interactions (N= number of fish in the sample).

**Species**	**Blue filament**		**Purple filament**		**Green filament**		**Red filament**		**White filament**		**Black filament**		**Total filament**	**Size (mm)**	**N**
C. undecimalis	**Mean ±SE**	**(%)**	**Mean ±SE**	**(%)**	**Mean ±SE**	**(%)**	**Mean ±SE**	**(%)**	**Mean ±SE**	**(%)**	**Mean ±SE**	**(%)**	**Mean ±SE**	**Mean ±SE**	
**Habitat**															
Upper	0.73 ±0.16	88		0		0	0.05 ±0.05	6	0.05 ±0.05	6		0	0.84 ±0.17	1.41 ±0.20	15
Middle	1.16 ±0.23	70	0.05±0.03	3	0.14 ±0.05	9	0.14 ±0.54	9	0.14 ±0.06	9	0.02 ±0.02	1	1.64 ±0.33	1.15 ±0.09	67
Lower	1.44 ±0.18	75	0.09 ±0.03	5	0.09 ±0.04	5	0.23 ±0.07	12	0.07 ±0.04	4		0	1.92 ±0.23	1.10 ±0.07	126
Coastal	1.14 ±0.18	75	0.10 ±0.04	7	0.08 ±0.03	6	0.12 ±0.03	8	0.07 ±0.02	5		0	1.49 ±0.21	1.08 ±0.05	190
**Season**															
Early Dry	1.14 ±0.19	75	0.12 ±0.05	8	0.06 ±0.03	4	0.12 ±0.06	8	0.06 ±.04	4	0.02 ±0.02	1	1.5 ±0.26	1.14 ±0.10	73
Late Dry	1.16 ±0.22	78	0.06 ±0.03	4	0.08 ±0.04	5	0.13 ±0.04	9	0.05 ±0.02	4		0	1.48 ±0.27	1.07 ±0.06	111
Early Rainy	1.30 ±0.17	73	0.02 ±0.02	1	0.13 ±0.4	7	0.19 ±0.06	11	0.12 ±0.04	7		0	1.72 ±0.22	1.09 ±0.07	136
Late Rainy	1.14 ±0.28	71	0.16 ±0.08	10	0.08 ±0.05	5	0.12 ±0.05	8	0.10 ±0.05	6		0	1.59 ±0.34	1.16 ±0.08	78
**Phase**															
Juvenile	1.16 ±0.14	77	0.02 ±0.02	1	0.12 ±0.05	8	0.18 ±0.06	12	0.02 ±0.02	1		0	1.52 ±0.17	1.10 ±0.10	75
Sub-adult	1.44 ±0.22	78	0.09 ±0.03	5	0.06 ±0.02	3	0.13 ±0.04	7	0.11 ±0.03	6		0	1.81 ±0.28	0.97 ±0.06	149
Adult	1.05 ±0.15	71	0.09 ±0.04	7	0.10 ±0.03	7	0.14 ±0.04	10	0.07 ±0.02	5	0.01 ±0.01	1	1.96 ±0.19	1.22 ±0.06	175

Supplementary Table 1. Continued.

Species	Blue filament		Purple filament		Green filament		Red filament		White filament		Black filament		Total filament	Size (mm)	
C. mexicanus	**Mean ±SE**	**(%)**	**Mean ±SE**	**(%)**	**Mean ±SE**	**(%)**	**Mean ±SE**	**(%)**	**Mean ±SE**	**(%)**	**Mean ±SE**	**(%)**	**Mean ±SE**	**Mean ±SE**	**N**
**Habitat**															
Upper	1.37 ±0.21	79	0.09 ±0.05	5	0.15 ±0.08	9		0	0.09 ±0.05	5	0.03 ±0.03	2	1.65 ±0.30	1.52 ±0.09	56
Middle	1.4 ±0.25	78	0.08 ±0.05	4	0.2 ±0.1	11	0.08 ±0.05	4	0.04 ±0.02	2		0	1.8 ±0.31	1.11 ±0.12	44
Lower	1.12 ±0.19	71	0.16 ±0.06	11	0.12 ±0.06	8	0.05 ±0.03	3	0.12 ±0.05	8		0	1.59 ±0.27	1.42 ±0.12	65
Coastal	0.97 ±0.13	84	0.04 ±0.03	4	0.04 ±0.02	4	0.08 ±0.02	7	0.01 ±0.01	1		0	1.16 ±0.15	1.0 ±0.06	100
**Season**															
Early Dry	0.5 ±0.31	73	0.06 ±0.06	9	0.06 ±0.06	9	0.06 ±0.06	9		0		0	0.69 ±0.35	0.84 ±0.15	10
Late Dry	1.33 ±0.27	82	0.05 ±0.05	4	0.05 ±0.05	4	0.05 ±0.05	4	0.05 ±0.05	4		0	1.55 ±0.33	1.17 ±0.16	28
Early Rainy	0.92 ±0.11	71	0.09 ±0.03	7	0.15 ±0.05	11	0.02 ±0.01	2	0.08 ±0.03	6	0.01 ±0.01	1	1.28 ±0.17	1.47 ±0.10	84
Late Rainy	1.37 ±0.16	79	0.09 ±0.04	5	0.08 ±0.03	5	0.09 ±0.03	5	0.04 ±0.02	2	0	0	1.71 ±0.20	1.11 ±0.06	130
**Phase**															
Juvenile	0.85 ±0.13	65	0.05 ±0.03	4	0.17 ±0.07	13	0.11 ±0.04	9	0.11 ±0.04	9		0	1.24 ±0.20	1.63 ±0.10	68
Sub-adult	1.19 ±0.12	80	0.08 ±0.02	6	0.09 ±0.03	6	0.07 ±0.02	5	0.04 ±0.02	3		0	1.49 ±0.15	1.07 ±0.06	140
Adult	1.28 ±0.26	80	0.14 ±0.07	9	0.02 ±0.02	2	0.11 ±0.05	7		0	0.03 ±0.02	2	1.6 ±0.33	1.15 ±0.09	57

Supplementary Table 1. Continued.

Species	Blue filament		Purple filament		Green filament		Red filament		White filament		Black filament		Total filament	Size (mm)	
C. pectinatus	Mean ±SE	(%)	Mean ±SE	(%)	Mean ±SE	(%)	Mean ±SE	(%)	Mean ±SE	(%)	Mean ±SE	(%)	Mean ±SE	Mean ±SE	N
**Habitat**															
Upper	0.28 ±0.28	100		0		0		0		0		0	0.28 ±0.28	0.33 ±0.10	2
Middle	0.7 ±0.26	88		0		0	0.1 ±0.11	13		0		0	0.8 ±0.29	1.16 ±0.30	8
Lower	1.2 ±0.37	75	0.2 ±0.2	13		0		0	0.2 ±0.2	13		0	1.6 ±0.51	1.63 ±0.44	7
Coastal	1 ±0.19	72	0.12 ±0.04	9	0.08 ±0.05	6	0.08 ±0.05	6	0.11 ±0.04	7		0	1.36 ±0.23	1.02 ±0.08	79
**Season**															
Early Dry	0.7 ±0.33	78	0.1 ±0.1	11		0	0.1 ±0.07	11		0		0	0.9 ±0.38	1.06 ±0.25	9
Late Dry	0.71 ±0.26	86		0		0	0.06 ±0.09	7	0.06 ±0.05	7		0	0.82 ±0.31	0.66 ±0.13	14
Early Rainy	1 ±0.38	74	0.13 ±0.09	10	0.08 ±0.07	6		0	0.13 ±0.07	10		0	1.30 ±0.43	1.03 ±0.16	31
Late Rainy	1 ±0.21	67	0.14 ±0.06	9	0.10 ±0.07	7	0.14 ±0.07	9	0.10 ±0.05	7		0	1.45 ±0.28	1.18 ±0.12	43
**Phase**															
Juvenile	0.31 ±0.17	100		0		0		0		0		0	0.31 ±0.08	1.43 ±0.74	3
Sub-adult	0.97 ±0.26	72	0.05 ±0.03	4	0.11 ±0.03	8	0.13 ±0.04	10	0.08 ±0.04	6		0	1.32 ±0.21	1.07 ±0.12	50
Adult	1.18 ±0.23	73	0.21 ±0.09	13	0.03 ±0.07	2	0.03 ±0.07	2	0.14 ±0.06	9		0	1.57 ±0.29	1.02 ±0.12	40

Supplementary Table 2.

Summary of the ANOVA for the length of microfilaments ingested by snooks in the Goiana Estuary, according to factors area [U (upper); M (middle); L (lower); C (coastal zone)], season [ED (early dry); LD (late dry); ER (early rainy); LR (late rainy)] and ontogeny [Juv (juveniles); Sub (sub-adults); Adu (adults)]. Bonferroni's test was used to determinate the sources of variances [F (F-values); df (degrees of freedom); *p*-value]. (ns: not significant) (**p* < 0.05).

		**Items in number**				
	**Factors**	**F**	***df***	***p*-value**		***Post-hoc***
C. undecimalis	Season	2.33	3	0.07	ns	
	Area	19.78	3	0.01	*	U
	Phase	24.04	2	0.01	*	Adu
	Season *vs*. Area	8.37	9	0.01	*	ER.L
	Season *vs*. Phase	7.67	6	0.01	*	LD.Sub
	Area *vs*. Phase	18.44	6	0.01	*	C.Ad
	Season *vs*. Area *vs*. Phase	3.82	18	0.01	*	ED.U.Juv
C. mexicanus	Season	77.26	3	0.01	*	ED
	Area	3.73	3	0.01	*	U
	Phase	22.50	2	0.01	*	Sub
	Season *vs*. Area	18.62	9	0.01	*	ER.C - LR.M - LR.C - ER.L - ER.U
	Season *vs*. Phase	11.05	6	0.01	*	LR.Sub
	Area *vs*. Phase	27.74	6	0.01	*	U.Juv
	Season *vs*. Area *vs*. Phase	12.62	18	0.01	*	ER.U.Ad - LR.U.Juv - ER.L.Juv
C. pectinatus	Season	9.81	3	0.01	*	ER - LR
	Area	78.58	3	0.01	*	C
	Phase	44.25	2	0.01	*	Juv
	Season *vs*. Area	16.90	9	0.01	*	LR.C
	Season *vs*. Phase	7.60	6	0.01	*	*
	Area *vs*. Phase	35.73	6	0.01	*	C.Ad - C.Sub
	Season *vs*. Area *vs*. Phase	8.46	18	0.01	*	ER.L.Sub

Supplementary Table 3.

Summary of the ANOVA for the colour of microfilaments in number ingested by *C. undecimalis* in the Goiana Estuary, according to factors area [U (upper); M (middle); L (lower); C (coastline)], season [ED (early dry); LD (late dry); ER (early rainy); LR (late rainy)] and ontogeny [Juv (juveniles); Sub (sub-adults); Adu (adults)]. Bonferroni's test was used to determinate the sources of variances [F (F-values); df (degrees of freedom); *p*-value]. (ns: not significant) (*$p < 0.05$).

		**Items in number**				
	**Factors**	**F**	***df***	***p*-value**		***Post-hoc***
Blue filaments	Season	2.55	3	0.06	ns	
	Area	6.40	3	0.01	*	U
	Phase	2.90	2	0.06	ns	
	Season *vs*. Area	4.32	9	0.01	*	ER.L
	Season *vs*. Phase	2.37	6	0.03	*	LD.Juv
	Area *vs*. Phase	3.66	6	0.01	*	C.Ad
	Season *vs*. Area *vs*. Phase	2.22	18	0.01	*	ER.L.Juv - ER.L.Sub
Purple filaments	Season	0.77	3	0.50	ns	
	Area	4.02	3	0.01	*	C
	Phase	3.82	2	0.02	*	Juv
	Season *vs*. Area	0.74	9	0.67	ns	
	Season *vs*. Phase	1.80	6	0.09	ns	
	Area *vs*. Phase	2.00	6	0.06	ns	
	Season *vs*. Area *vs*. Phase	1.11	18	0.34	ns	
Green filaments	Season	2.42	3	0.06	ns	
	Area	3.11	3	0.02	*	U
	Phase	0.90	2	0.40	ns	
	Season *vs*. Area	1.74	9	0.08	ns	
	Season *vs*. Phase	1.68	6	0.12	ns	
	Area *vs*. Phase	1.45	6	0.19	ns	
	Season *vs*. Area *vs*. Phase	1.20	18	0.05	*	ER.M.Sub - ER.M.Ad - ED.C.Juv - LD.C.Ad - ER.C.Ad

Supplementary Table 3. Continued.

		Items in number				
	**Factors**	**F**	***df***	***p*-value**		***Post-hoc***
Red filaments	Season	2.30	3	0.07	ns	
	Area	3.88	3	0.01	*	U
	Phase	0.87	2	0.41	ns	
	Season *vs*. Area	1.43	9	0.17	ns	
	Season *vs*. Phase	1.80	6	0.09	ns	
	Area *vs*. Phase	1.11	6	0.35	ns	
	Season *vs*. Area *vs*. Phase	1.22	18	0.24	ns	
White filaments	Season	1.19	3	0.31	ns	
	Area	1.28	3	0.27	ns	
	Phase	3.12	2	0.04	*	Juv
	Season *vs*. Area	1.11	9	0.35	ns	
	Season *vs*. Phase	1.27	6	0.26	ns	
	Area *vs*. Phase	1.77	6	0.10	ns	
	Season *vs*. Area *vs*. Phase	1.32	18	0.17	ns	

Supplementary Table 4.

Summary of the ANOVA for the colour of microfilaments in number ingested by *C. mexicanus* in the Goiana Estuary, according to factors area [U (upper); M (middle); L (lower); C (coastal zone)], season [ED (early dry); LD (late dry); ER (early rainy); LR (late rainy)] and ontogeny [Juv (juveniles); Sub (sub-adults); Adu (adults)]. Bonferroni's test was used to determinate the sources of variances [F (F-values); df (degrees of freedom); *p*-value]. (ns: not significant) (*$p < 0.05$).

		**Items in number**				
	**Factors**	**F**	***df***	***p*-value**		***Post-hoc***
Blue filaments	Season	51.44	3	0.01	*	LR
	Area	0.24	3	0.86	ns	
	Phase	43.74	2	0.01	*	Sub
	Season *vs*. Area	10.04	9	0.01	*	ER.U - LR.M -LD.M - LR.C - ER.L - LR.U
	Season *vs*. Phase	12.34	6	0.01	*	LR.Sub
	Area *vs*. Phase	14.21	6	0.01	*	L.Sub
	Season *vs*. Area *vs*. Phase	6.37	18	0.01	*	LD.M.Sub - LR.C.Sub
Purple filaments	Season	2.55	3	0.06	ns	
	Area	0.82	3	0.48	ns	
	Phase	1.28	2	0.27	ns	
	Season *vs*. Area	2.24	9	0.02	*	ER.L
	Season *vs*. Phase	0.73	6	0.62	ns	
	Area *vs*. Phase	2.07	6	0.06	ns	
	Season *vs*. Area *vs*. Phase	1.7	18	0.04	*	ER.L.Sub
Green filaments	Season	3.25	3	0.02	*	ER
	Area	0.20	3	0.88	ns	
	Phase	2.52	2	0.08	ns	
	Season *vs*. Area	1.91	9	0.06	ns	
	Season *vs*. Phase	0.68	6	0.66	ns	
	Area *vs*. Phase	1.92	6	0.07	ns	
	Season *vs*. Area *vs*. Phase	0.85	18	0.63	ns	

Supplementary Table 4. Continued.

	Factors	Items in number F	*df*	*p*-value		*Post-hoc*
Red filaments	Season	1.36	3	0.25	ns	
	Area	5.17	3	0.01	*	U
	Phase	0.92	2	0.39	ns	
	Season *vs*. Area	0.53	9	0.84	ns	
	Season *vs*. Phase	1.05	6	0.38	ns	
	Area *vs*. Phase	1.71	6	0.11	ns	
	Season *vs*. Area *vs*. Phase	0.76	18	0.74	ns	
White filaments	Season	3.39	3	0.01	*	ED LD ER LR
	Area	2.64	3	0.06	ns	
	Phase	3.84	2	0.02	*	Adu
	Season *vs*. Area	1.92	9	0.06	ns	
	Season *vs*. Phase	3.47	6	0.01	*	ER.Juv
	Area *vs*. Phase	2.47	6	0.02	*	U.Juv - L.Sub
	Season *vs*. Area *vs*. Phase	3.22	18	0.01	*	LR.L.Sub

Supplementary Table 5.
Summary of the ANOVA for the colour of microfilaments in number ingested by *C. pectinatus* in the Goiana Estuary, according to factors area [U (upper); M (middle); L (lower); C (coastal zone)], season [ED (early dry); LD (late dry); ER (early rainy); LR (late rainy)] and ontogeny [Juv (juveniles); Sub (sub-adults); Adu (adults)]. Bonferroni's test was used to determinate the sources of variances [F (F-values); df (degrees of freedom); *p*-value]. (ns: not significant) (**p* < 0.05).

		Items in number				
	**Factors**	**F**	***df***	***p*-value**		***Post-hoc***
Blue filaments	Season	4.16	3	0.01	*	LR
	Area	23.91	3	0.01	*	C
	Phase	8.18	2	0.01	*	Juv
	Season *vs*. Area	3.89	9	0.01	*	LR.C
	Season *vs*. Phase	0.40	6	0.87	ns	LR.Sub
	Area *vs*. Phase	10.97	6	0.01	*	C.Ad - C.Sub
	Season *vs*. Area *vs*. Phase	1.92	18	0.01	*	LR.C.Sub - LR.C.Ad
Purple filaments	Season	2.18	3	0.09	ns	
	Area	7.52	3	0.01	*	C
	Phase	5.00	2	0.01	*	Adu
	Season *vs*. Area	2.03	9	0.03	*	LR.C
	Season *vs*. Phase	0.99	6	0.42	ns	
	Area *vs*. Phase	6.06	6	0.01	*	C.Ad
	Season *vs*. Area *vs*. Phase	1.26	18	0.21	ns	
Green filaments	Season	1.46	3	0.22	ns	
	Area	4.34	3	0.01	*	C
	Phase	1.79	2	0.16	ns	
	Season *vs*. Area	1.46	9	0.16	ns	
	Season *vs*. Phase	1.07	6	0.37	ns	
	Area *vs*. Phase	1.79	6	0.10	ns	
	Season *vs*. Area *vs*. Phase	1.07	18	0.37	ns	

Supplementary Table 5. Continued.

	Factors	Items in number F	*df*	*p*-value		*Post-hoc*
Red filaments	Season	0.66	3	0.57	ns	
	Area	2.14	3	0.09	ns	
	Phase	1.85	2	0.15	ns	
	Season *vs*. Area	1.43	9	0.17	ns	
	Season *vs*. Phase	1.52	6	0.17	ns	
	Area *vs*. Phase	0.79	6	0.57	ns	
	Season *vs*. Area *vs*. Phase	1.08	18	0.35	ns	
White filaments	Season	1.74	3	0.15	ns	
	Area	6.04	3	0.01	*	C
	Phase	2.80	2	0.06	ns	
	Season *vs*. Area	1.13	9	0.33	ns	
	Season *vs*. Phase	0.77	6	0.58	ns	
	Area *vs*. Phase	3.16	6	0.01	*	C.Ad
	Season *vs*. Area *vs*. Phase	0.58	18	0.90	ns	

Supplementary Table 6.
Food items ingested by snooks grouped into ecologic/taxonomic categories.

Food items Groups			
**Pelagic fishes**	Engraulidae<br>*Anchovia clupeoides*<br>*Cetengraulis edentulus*<br>Clupeidae<br>*Rhinosardinia bahiensis*<br>*Harengula clupeola*<br>*Opisthonema oglinum*<br>*Odontognathus mucronatus*<br>*Lycengraulis grossidens*<br>*Mugil liza*<br>*Caranx latus*<br>*Trichiurus lepturus*<br>*Hemiramphus brasiliensis*	**Demersal fishes**	*Cathorops spixii*<br>*Achirus lineatus*<br>*Cynoscion acoupa*<br>*Stellifer stellifer*<br>*Stellifer rastrifer*<br>*Stellifer brasiliensis*<br>*Menticirrhus littoralis*<br>*Centropomus undecimalis*<br>*Pomadasys corvinaeformis*<br>*Diapterus rhombeus*<br>*Eucinostomus melanopterus*<br>*Eugerres brasilianus*<br>Gobidae<br>*Bathygobius soporator*<br>*Eleotris pisonis*<br>*Myrophis punctatus*
**Macrocrustaceans**	Paguridae<br>Penaeidae shrimp<br>*Callinectes danae*<br>*Aratus pisonii*<br>*Ucides cordatus*	**Microcrustaceans**	Amphipoda<br>Mysidacea<br>Copepoda<br>Isopoda
**Bristle worms**	Nereidae<br>Syllidae	**Organic matter**	Mangrove fragments<br>Seaweed

Supplementary Table 7.

Summary of the ANOVA for the weight of food groups ingested by *C. mexicanus* in the Goiana Estuary, according to factors area [U (upper); M (middle); L (lower); C (coastal zone)], season [ED (early dry); LD (late dry); ER (early rainy); LR (late rainy)] and ontogeny [Juv (juveniles); Sub (sub-adults); Adu (adults)]. Bonferroni's test was used to determinate the sources of variances [F (F-values); df (degrees of freedom); *p*-value]. (ns: not significant) (*$p < 0.05$).

		Items in number				
	**Factors**	**F**	***df***	***p*-value**		***Post-hoc***
Pelagic fishes	Season	4.79	3	0.01	*	ED
	Area	19.74	3	0.01	*	C
	Phase	14.15	2	0.01	*	Juv
	Season *vs*. Area	8.07	9	0.01	*	ER.C - LR.C
	Season *vs*. Phase	1.67	6	0.12	ns	
	Area *vs*. Phase	6.88	6	0.01	*	C.Sub - C.Ad
	Season *vs*. Area *vs*. Phase	4.29	18	0.01	*	ER.C.Sub - LR.C.Ad
Demersal fishes	Season	0.08	3	0.96	ns	
	Area	1.33	3	0.26	ns	
	Phase	0.93	2	0.39	ns	
	Season *vs*. Area	0.71	9	0.69	ns	
	Season *vs*. Phase	0.76	6	0.59	ns	
	Area *vs*. Phase	0.96	6	0.44	ns	
	Season *vs*. Area *vs*. Phase	1.09	18	0.35	ns	
Macrocrustaceans	Season	5.56	3	0.01	*	ER
	Area	1.66	3	0.17	ns	
	Phase	0.37	2	0.68	ns	
	Season *vs*. Area	1.14	9	0.33	ns	
	Season *vs*. Phase	0.76	6	0.59	ns	
	Area *vs*. Phase	2.96	6	0.01	*	C.Sub
	Season *vs*. Area *vs*. Phase	1.37	18	0.14	ns	
Microcrustaceans	Season	1.20	3	0.30	ns	
	Area	1.11	3	0.34	ns	
	Phase	1.16	2	0.31	ns	
	Season *vs*. Area	0.78	9	0.63	ns	
	Season *vs*. Phase	1.47	6	0.18	ns	
	Area *vs*. Phase	0.72	6	0.63	ns	
	Season *vs*. Area *vs*. Phase	0.91	18	0.55	ns	

Supplementary Table 7. Continued.

	Factors	Items in number F	*df*	*p*-value		*Post-hoc*
Bristle worms	Season	6.16	3	0.01	*	ER
	Area	5.96	3	0.01	*	L
	Phase	2.09	2	0.12	ns	
	Season *vs*. Area	7.87	9	0.01	*	ER.C
	Season *vs*. Phase	2.19	6	0.04	*	ER.Juv
	Area *vs*. Phase	2.11	6	0.06	ns	
	Season *vs*. Area *vs*. Phase	2.11	18	0.01	*	ER.L.Juv
Organic matter	Season	1.97	3	0.11	ns	
	Area	1.90	3	0.12	ns	
	Phase	1.43	2	0.24	ns	
	Season *vs*. Area	2.63	9	0.01	*	ER.L
	Season *vs*. Phase	2.49	6	0.02	*	ER.Juv
	Area *vs*. Phase	2.57	6	0.02	*	L.Juv
	Season *vs*. Area *vs*. Phase	2.18	18	0.01	*	ER.L.Juv

Supplementary Table 8.

Summary of the ANOVA for the weight of food groups ingested by *C. undecimalis* in the Goiana Estuary, according to factors area [U (upper); M (middle); L (lower); C (coastal zone)], season [ED (early dry); LD (late dry); ER (early rainy); LR (late rainy)] and ontogeny [Juv (juveniles); Sub (sub-adults); Adu (adults)]. Bonferroni's test was used to determinate the sources of variances [F (F-values); df (degrees of freedom); *p*-value]. (ns: not significant) (**p* < 0.05).

		**Items in number**				
	**Factors**	**F**	***df***	***p*-value**		***Post-hoc***
Pelagic fishes	Season	1.75	3	0.15	ns	
	Area	34.07	3	0.01	*	C
	Phase	14.24	2	0.01	*	Adu
	Season *vs*. Area	2.06	9	0.03	*	ED.C - LD.C
	Season *vs*. Phase	1.88	6	0.08	ns	
	Area *vs*. Phase	4.80	6	0.01	*	C.Ad
	Season *vs*. Area *vs*. Phase	1.78	18	0.02	*	ED.C.Ad - LD.C.Ad - ER.C.Ad - LR.C.Ad - LD.C.Sub
Demersal fishes	Season	1.16	3	0.32	ns	
	Area	9.46	3	0.01	*	U
	Phase	12.91	2	0.01	*	Adu
	Season *vs*. Area	3.21	9	0.01	*	LD.M
	Season *vs*. Phase	1.66	6	0.13	ns	
	Area *vs*. Phase	6.87	6	0.01	*	C.Ad
	Season *vs*. Area *vs*. Phase	1.41	18	0.12	ns	
Macrocrustaceans	Season	0.21	3	0.88	ns	
	Area	2.57	3	0.06	ns	
	Phase	3.47	2	0.03	*	Sub
	Season *vs*. Area	3.01	9	0.01	*	LR.C
	Season *vs*. Phase	1.10	6	0.35	ns	
	Area *vs*. Phase	2.36	6	0.03	*	C.Ad
	Season *vs*. Area *vs*. Phase	1.34	18	0.16	ns	
Microcrustaceans	Season	0.94	3	0.41	ns	
	Area	1.10	3	0.34	ns	
	Phase	0.92	2	0.39	ns	
	Season *vs*. Area	0.96	9	0.47	ns	
	Season *vs*. Phase	1.02	6	0.40	ns	
	Area *vs*. Phase	0.94	6	0.46	ns	
	Season *vs*. Area *vs*. Phase	1.01	18	0.44	ns	

Supplementary Table 8. Continued.

	Factors	Items in number F	*df*	*p*-value		*Post-hoc*
Bristle worms	Season	1.11	3	0.34	ns	
	Area	1.93	3	0.12	ns	
	Phase	0.53	2	0.58	ns	
	Season *vs*. Area	2.40	9	0.01	*	ED.U
	Season *vs*. Phase	0.76	6	0.59	ns	
	Area *vs*. Phase	0.36	6	0.90	ns	
	Season *vs*. Area *vs*. Phase	0.82	18	0.67	ns	
Organic matter	Season	2.56	3	0.06	ns	
	Area	2.94	3	0.03	*	L
	Phase	1.06	2	0.34	ns	
	Season *vs*. Area	0.56	9	0.82	ns	
	Season *vs*. Phase	3.05	6	0.01	*	LD.Ad
	Area *vs*. Phase	0.59	6	0.73	ns	
	Season *vs*. Area *vs*. Phase	1.30	18	0.19	ns	

Supplementary Table 9.

Summary of the ANOVA for the weight of food groups ingested by *C. pectinatus* in the Goiana Estuary, according to factors area [U (upper); M (middle); L (lower); C (coastal zone)], season [ED (early dry); LD (late dry); ER (early rainy); LR (late rainy)] and ontogeny [Juv (juveniles); Sub (sub-adults); Adu (adults)]. Bonferroni's test was used to determinate the sources of variances [F (F-values); df (degrees of freedom); *p*-value]. (ns: not significant) (*$p < 0.05$).

		**Items in number**				
	**Factors**	**F**	***df***	***p*-value**		***Post-hoc***
Pelagic fishes	Season	1.57	3	0.19	ns	
	Area	25.15	3	0.01	*	C
	Phase	9.46	2	0.01	*	Adu
	Season *vs*. Area	1.57	9	0.12	ns	
	Season *vs*. Phase	1.45	6	0.19	ns	
	Area *vs*. Phase	9.46	6	0.01	*	C.Sub - C.Ad
	Season *vs*. Area *vs*. Phase	1.45	18	0.11	ns	
Demersal fishes	Season	1.60	3	0.18	ns	
	Area	2.87	3	0.03	*	C
	Phase	0.64	2	0.52	ns	
	Season *vs*. Area	1.47	9	0.15	ns	
	Season *vs*. Phase	2.13	6	0.06	ns	
	Area *vs*. Phase	1.49	6	0.18	ns	
	Season *vs*. Area *vs*. Phase	2.19	18	0.01	*	ER.C.Ad
Macrocrustaceans	Season	1.52	3	0.20	ns	
	Area	3.90	3	0.01	*	C
	Phase	2.73	2	0.06	ns	
	Season *vs*. Area	2.65	9	0.01	*	ER.C
	Season *vs*. Phase	1.73	6	0.11	ns	
	Area *vs*. Phase	2.31	6	0.03	*	C.Sub
	Season *vs*. Area *vs*. Phase	2.31	18	0.01	*	LR.C.Sub
Microcrustaceans	Season	0.85	3	0.46	ns	
	Area	1.46	3	0.22	ns	
	Phase	1.05	2	0.34	ns	
	Season *vs*. Area	0.70	9	0.70	ns	
	Season *vs*. Phase	0.74	6	0.61	ns	
	Area *vs*. Phase	0.59	6	0.73	ns	
	Season *vs*. Area *vs*. Phase	1.22	18	0.24	ns	

Supplementary Table 9. Continued.

	Factors	Items in number F	*df*	*p*-value		*Post-hoc*
Bristle worms	Season	2.39	3	0.06	ns	
	Area	2.51	3	0.06	ns	
	Phase	1.43	2	0.24	ns	
	Season *vs*. Area	3.41	9	0.01	*	ER.L
	Season *vs*. Phase	0.91	6	0.48	ns	
	Area *vs*. Phase	1.00	6	0.42	ns	
	Season *vs*. Area *vs*. Phase	1.02	18	0.43	ns	
Organic matter	Season	2.54	3	0.06	ns	
	Area	3.51	3	0.01	*	C
	Phase	1.92	2	0.14	ns	
	Season *vs*. Area	2.59	9	0.01	*	LR.C
	Season *vs*. Phase	3.06	6	0.01	*	LR.Sub
	Area *vs*. Phase	2.58	6	0.01	*	C.Sub
	Season *vs*. Area *vs*. Phase	3.06	18	0.01	*	LR.C.Sub

Supplementary Table 10.

Summary of the Canonical Correspondence Analysis (CCA) using six environmental variables (water temperature, Secchi depth, salinity, dissolved oxygen and rainfall) and the index of relative importance (I_{RI}) for the main food items ingested by snooks according to the factors habitats, seasons and ontogenetic phases.

Statistics	Axis I	Axis II	Environmental variables	*p*-value
Eigenvalue	0.095	0.031	Salinity	0.01
Pseudo-canonical correlation %	55	36.4	Dissolved oxygen (mg l^{-1})	0.01
			Secchi depth (cm)	0.01
Explained fitted variation of species-environmental variables %	61.8	19.5	Water temperature (ºC)	0.95
			Rainfall (mm)	0.95

Supplementary Table 11

Size classes of different ontogenetic phases of snooks.

	**Juvenile**	**Sub-adult**	**Adult**
C. undecimalis	< 263 mm	263 - 454 mm	> 454 mm
C. mexicanus	< 213 mm	213 - 361 mm	> 361 mm
C. pectinatus	< 146 mm	146 - 261 mm	> 261 mm

REFERENCES

1. Barletta, M. & Costa, M. F. Living and Non-living Resources Exploitation in a Tropical Semi-arid Estuary. *J. Coast. Res.* **2009**, 371–375 (2009).

2. Barletta, M., Lima, A. R. A., Costa, M. F. & Dantas, D. V. Estuarine Ecoclines and the Associated Fauna: Ecological Information as the Basis for Ecosystem Conservation. in *Coastal Wetlands: Alteration and Remediation* (eds. Finkl, C. W. & Makowski, C.) **21**, 479–512 (Springer International Publishing, 2017).

3. Ferreira, G. V. B. *et al.* Plastic debris contamination in the life cycle of Acoupa weakfish (*Cynoscion acoupa*) in a tropical estuary. *ICES J. Mar. Sci. J. du Cons.* **73**, 2695–2707 (2016).

4. Ruppert, E. E., Fox, S. R. & Barnes, D. R. *Invertebrate zoology: a functional evolutionary approach*. (Thomson Brooks/Cole, 2004).

5. Menezes, N. A. & Figueiredo, J. L. *Manual de peixes marinhos do sudeste do Brasil IV.Teleostei (3)*. (Museu de Zoologia, Universidade de São Paulo, 1980).

6. Box, G. E. P. & Cox, D. An Analysis of Transformations. *J. R. Stat. Soc.* **26**, 211–252 (1964).

7. Underwood, A. J. *Experiments in ecology: their logical design and interpretation using analysis of variance*. (Cambridge University Press, 1997).

8. Quinn, R. & Keough, M. *Experimental design and data analysis for biologists*. (Cambridge University Press, 2002).

9. Ter Braak, C. J. F. & Smilauer, P. CANOCO References Manual and CanocoDraw for Windows User's Guide: Software for Canonical Community Ordination (Version 4.5). 500 (2002). Available at: www.canoco.com.

10. Hyslop, E. J. Stomach contents analysis: A review of methods and their application. *J. Fish Biol.* **17**, 411–429 (1980).
